# Multidisciplinary Tracking of Highly Pathogenic Avian Influenza A(H5N1) Outbreak in Griffon Vultures, Southern Europe, 2022

**DOI:** 10.3201/eid3108.241456

**Published:** 2025-08

**Authors:** Julien Hirschinger, Ursula Höfle, Alberto Sánchez-Cano, Claire Guinat, Guillaume Croville, Marta Barral, José Antonio Donázar, Chloé Le Gall Ladevèze, Mathilda Walch, Vega Alvarez, Xeider Gerrikagoitia, Louis Du Plessis, Simon Dellicour, Eneko Arrondo, José Antonio Sánchez-Zapata, Ainara Cortés-Avizanda, Sara Minayo Martín, Jérémy Tornos, Samuel Perret, Thierry Boulinier, Pascal Orabi, Anne Van De Wiele, Jean-Luc Guerin, Olivier Duriez, Guillaume Le Loc’h

**Affiliations:** IHAP, ENVT, INRAE, Université de Toulouse, Toulouse, France (J. Hirschinger, C. Guinat, G. Croville, C. Le Gall Ladevèze, M. Walch, J.-L. Guerin, G. Le Loc’h); Grupo SaBio, Instituto de Investigación en Recursos Cinegéticos (CSIC-UCLM-JCCM), Ciudad Real, Spain (U. Höfle, A. Sánchez-Cano, S. Minayo Martín); NEIKER–Basque Institute for Agricultural Research and Development, Basque Research and Technology Alliance (BRTA), Derio, Spain (M. Barral, V. Alvarez, X. Gerrikagoitia); Estación Biológica de Doñana, CSIC, Sevilla, Spain (J.A. Donázar, A. Cortés-Avizanda); ETH Zurich, Basel, Switzerland (L. Du Plessis); Swiss Institute of Bioinformatics, Lausanne, Switzerland (L. Du Plessis); Spatial Epidemiology Lab, Université Libre de Bruxelles, Brussels, Belgium (S. Dellicour); Rega Institute, KU Leuven, Leuven, Belgium (S. Dellicour); Universidad Miguel Hernández, Elche, Spain (J.A. Sánchez-Zapata); Centre d’Ecologie Fonctionnelle et Evolutive, Univ Montpellier, CNRS, EPHE, IRD, Montpellier, France (J. Tornos, S. Perret, T. Boulinier, O. Duriez); Ligue pour la Protection des Oiseaux, Rochefort, France (P. Orabi); Office Français de la Biodiversité, Vincennes, France (A. Van De Wiele); University of Granada, Granada, Spain (E. Arrondo)

**Keywords:** highly pathogenic avian influenza A, viruses, respiratory infections, zoonoses, H5N1, clade 2.3.4.4b, Griffon vultures, *Gyps fulvus*, virology, serology, phylogeny, ecology, southern Europe, Spain, France

## Abstract

Since 2021, highly pathogenic avian influenza (HPAI) A(H5N1) clade 2.3.4.4b virus has affected wild bird populations globally. Griffon vultures (*Gyps fulvus*), a species hitherto considered unexposed, experienced an HPAI H5N1 outbreak in 2022 in southern Europe, leading to moderate mortality and reduced breeding success. The integration of virological, serologic, phylogenetic, and ecologic data revealed a short yet intense viral circulation and a probable common source of infection. The dissemination across Spain and France was likely caused by frequent interpopulation movements of birds. This integrated overview of the 2022 HPAI outbreak in vultures provides novel insights into the role of large-scale movements of wild birds in the spread of such disease. Understanding the epidemiologic dynamics of HPAI H5N1 in these scavenger species is crucial because the birds play vital roles in ecosystem functioning. Their susceptibility to this virus highlights potential broader ecologic effects of the ongoing outbreaks.

Since 2021, highly pathogenic avian influenza (HPAI) A(H5N1) clade 2.3.4.4b virus has emerged as a devastating pathogen in terms of bird species diversity, abundance, geographic extent, and economic losses (*1*). Although the effects on domestic birds have been staggering at >500 million reported deaths, the full extent of the toll on wild birds is unknown (*2*). Approximately 420,000 wild bird deaths have been reported, likely a considerable underestimate (*3*), and the diversity and number of affected species imply a profound threat to biodiversity (*4*).

This ongoing panzootic represents a paradigm shift in H5Nx avian influenza. HPAI H5N1 infections have now been reported on all continents except Oceania and in >50 mammal species (*5*,*6*). Most mammal infections have been reported in predators and scavengers, but livestock have also been affected, notably cattle in the United States (*7*). Furthermore, the loss of traditional seasonality, evidenced by outbreaks now persisting year-round, also represents a profound shift in HPAI virus ecology (*8*,*9*). Evidence for sustained mammal-to-mammal transmission is inconclusive, but the unprecedented geographic spread of the virus, coupled with the number of species infected, has raised concerns about the ability of the virus to expand its host range and gain pandemic potential (*10*).

Among wild birds, gregarious species, particularly those with colonial nesting behavior, have shown heightened vulnerability (*11*). Colonies of seabirds have experienced exceptionally high mortality rates worldwide (*12*–*16*). This susceptibility is likely because of enhanced virus transmission within densely aggregated avian populations, where the proximity of birds contributes to the rapid spread of the virus.

Vultures have been considered relatively resilient to pathogens, including HPAI virus, because of their scavenging diet and their associated physiologic adaptations to cope with pathogens (*17*). Before the current panzootic, only a local outbreak of HPAI had been reported in hooded vultures (*Necrosyrtes monachus*) in Burkina Faso in 2006 (*18*). Similarly, although ornitophagous raptors and scavengers have previously been only sporadically affected by HPAI H5Nx, they have been unexpectedly affected during the ongoing panzootic; birds affected have included obligate or occasional scavengers such as bald eagles (*Haliaaetus leucocephalus*), black vultures (*Coragyps atratus*), and California condors (*Gymnogyps californianus*) in North America and griffon vultures (*Gyps fulvus*) in Europe (*19*,*20*).

In spring 2022, abnormal deaths of nestling and adult griffon vultures were detected in Spain and France; HPAI H5N1 infection was confirmed by quantitative reverse transcription PCR (qRT-PCR) (Appendix Figure 1). This occurrence prompted an investigation into the epidemiology of HPAI in this vulture population. Leveraging ongoing ecologic studies, we investigated the origin of infection and assessed the nature and magnitude of viral spread through an integrated analysis of virological, serologic, genomic, and ecologic data obtained from field sampling in France and Spain. In addition to seroepidemiology and viral phylogenetic approaches, we further used long-term global positioning system (GPS) tracking data to evaluate potential sources of exposure, to study population connectivity pathways, and to investigate the potential of long-range contamination by movements of infected animals.

## Methods

We collated and jointly analyzed information from griffon vultures collected from Spain and France in the framework of several research and surveillance programs dedicated to the study of vulture ecology, population dynamics, or HPAI outbreak follow-up (Appendix). In addition, the sampling included birds from national and local surveillance programs or those submitted for diagnosis to the institutions of the authors. In Spain, griffon vulture captures were performed in numerous colonies across the Iberian Peninsula during 2020–2022. Adult griffon vultures were captured with net traps or walk-in traps at vulture feeding stations, and nestlings were captured at nests. In France, 3 capture sessions were performed in 2022 and 2023 using walk-in traps at 5 sites.

All birds were ringed and a subset of vultures were fitted with GPS satellite transmitters. Blood samples, as well as oropharyngeal and cloacal swab samples, were collected from live birds. From dead birds, vascular feathers and tissues from the main organs (spleen, pancreas, heart, brain, trachea, intestine, lungs, and liver) were collected.

We extracted nucleic acids and submitted them to generic matrix gene qRT-PCR. We then submitted positive samples for nanopore sequencing (Oxford Nanopore Technologies, https://nanoporetech.com) and used consensus genomes in molecular marker and phylogeographic analysis.

We separated serum from the cell pellet by centrifugation in blood samples and stored at −20°C until analysis. We inactivated and tested serum samples with commercial ELISA and submitted positive samples to a hemagglutination inhibition (HI) test. We categorized HI samples as positive for an antigen if the titer was >16. We then estimated true seroprevalence by using the epidemiologic calculator Epitools (https://epitools.ausvet.com.au/prevalence).

We examined movement patterns of GPS-tagged griffon vultures during the 4-month period from March 1–June 29 during 2022 (outbreak year) and 2023 (control year), characterizing tracks as local or transit movements or immobility. We calculated daily distances traveled (DDT) and used generalized linear mixed models to identify factors affecting DDT. We first examined the movement patterns of a large sample of 114 birds, then examined the spatial and temporal dimensions of the movements of a subsample of 16 birds that remained in Europe during the study period. 

## Results

### HPAI H5N1 Outbreak Dynamics

**Figure 1:**
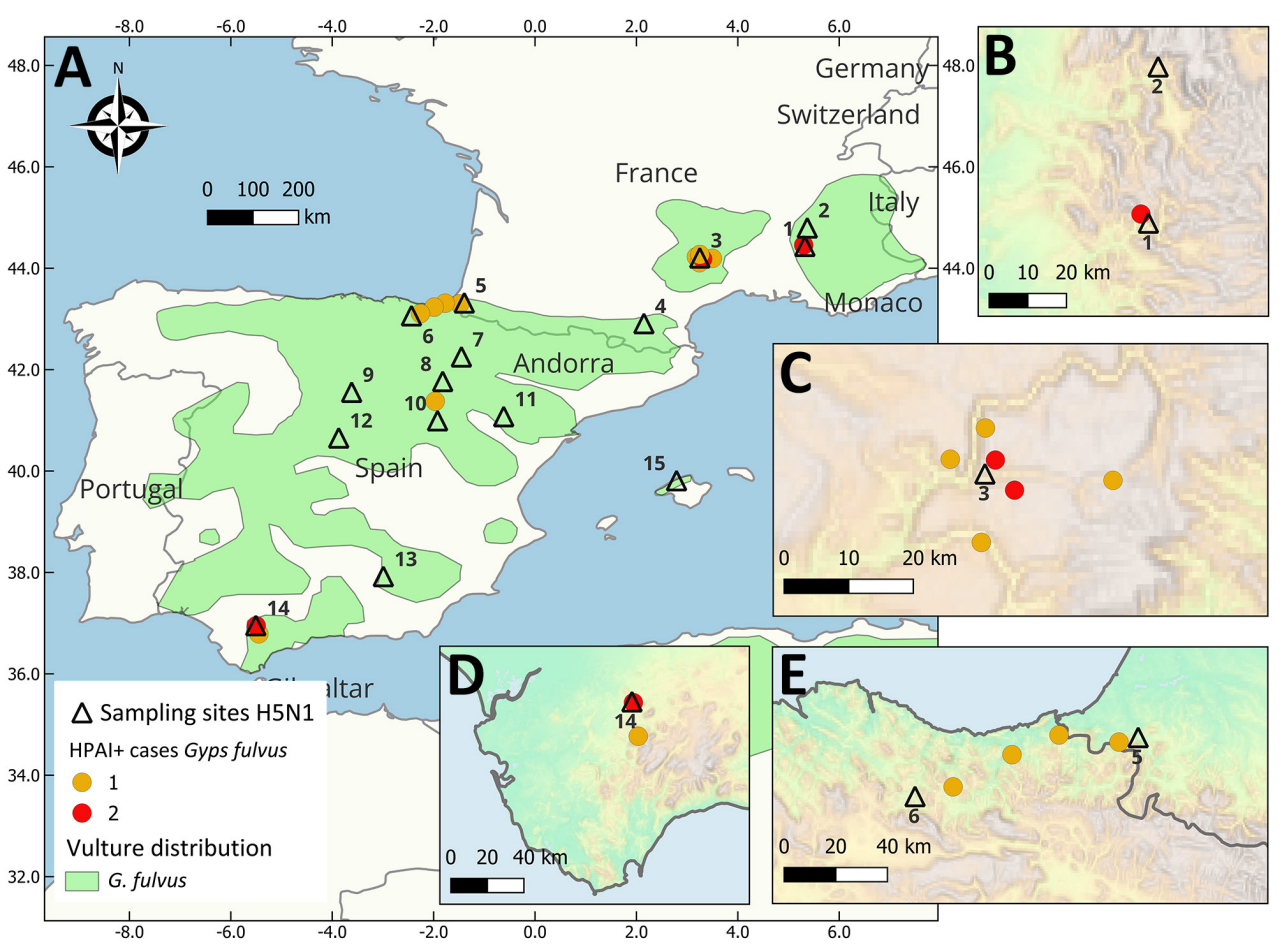
Sampling sites of live vultures (black triangles) and collection sites of carcasses of confirmed HPAI-positive vultures (dots) during March 2020–August 2023 in study of multidisciplinary tracking of highly pathogenic avian influenza A(H5N1) outbreak in griffon vultures (*Gyps fulvus*), southern Europe, 2022. The correspondence between site identification and the details of site names, region, or province area are given in the Appendix. The color of collection sites indicates the number of cases in each site. A) Sampling sites in France and Spain; light green shaded areas show the breeding range of Griffon vultures. Insets provide details of specific sites: B) Alps, C) Massif Central, D) South Spain, and E) Pyrenees and North Spain.

During April–August 2022, a total of 5 griffon vultures in Spain and 11 in France were confirmed to have died from HPAI H5N1 infection (Figure 1; Appendix). Despite the advanced state of decomposition, severe generalized congestion was detected on postmortem examination.

Apparently healthy live vultures sampled in both countries during March 2020–August 2023 tested negative for avian influenza virus (AIV) in oropharyngeal and cloacal swab samples (France, n = 393; Spain, n = 216). However, 2 birds admitted to a rehabilitation center in northern Spain (Figure 1, site 6) tested positive for HPAI H5 on May 5, 2022, and May 11, 2022. Both birds displayed weakness and severe central nervous system symptoms, including torticollis and inability to fly (Appendix). Only 1 of 87 live griffon vulture nestlings from Spain for which vascular feathers were available tested positive for HPAI H5 on May 24, 2022. The nestling, from southern Spain (site 14), lacked clinical signs at the time of ringing but was found dead on June 10, 2022; the remains tested positive for HPAI H5 (Appendix).

Exposure to H5 AIV in flying vultures was confirmed by the detection of H5-specific antibodies by HI tests after ELISA screening in 58 of 392 birds in France and 7 of 51 birds in Spain captured after the outbreak (summer 2022, autumn 2022, and summer 2023) (Table; Appendix Table 1). However, only 3 of 51 nestlings tested in Spain after the outbreak (summer 2022 and summer 2023) had H5-specific antibodies. Of 17 flying birds captured in Spain before the outbreak in spring 2020, a total of 8 were seropositive against strains of AIV other than H5 or H7, whereas none of the 50 nestlings tested before the outbreak (summer 2021) were seropositive against AIV. Of 128 H5 ELISA-positive vultures in France, 70 could not be confirmed by H5-specific HI tests.

**Table:** Results of ELISA tests and H5-HI test of ELISA-positive serum samples in study of multidisciplinary tracking of highly pathogenic avian influenza A(H5N1) outbreak in griffon vultures, southern Europe, 2022*

Country	Date	ELISA test			H5-HI test
		Type	Estimated prevalence, % (95% CI)		Estimated prevalence, % (95% CI)
France	2020	NA	NA		NA
	2021	NA	NA		NA
	2022	Anti H5	28 (22–36)		82 (69–92)
	2023	Anti H5	20 (12–28)		20 (9–37)
Spain	2020	Anti AIV	47 (25–71)		0 (0–37)
	2021	Anti AIV	0 (0–5)		NA
	2022	Anti AIV	16 (9–26)		38 (17–68)
	2023	Anti AIV	31 (13–56)		100 (66–100)

*For details see Appendix Table 1). AIV, avian influenza virus; H5-HI, H5 hemagglutination inhibition; NA, not applicable.

### Origin and Spatial Spread of Infection

We performed phylogenetic analysis on a dataset of 12 hemagglutinin genetic sequences retrieved from HPAI H5N1–infected griffon vultures (8 generated in this study), together with a background dataset of sequences from poultry and wild birds that were sampled in Europe during November 8, 2021–September 1, 2022 (n = 571) (Appendix). The analysis confirmed that the sequences obtained in this study belong to clade 2.3.4.4b and showed that most vulture sequences were grouped into a distinct clade (n = 32) in a maximum-likelihood tree (bootstrap support = 0.83) (Appendix), with vulture sequences already identified as genotype AB (*21*). Mutation analysis of the vulture sequences showed that several variable sites were identified in the hemagglutinin segment, some of which have been previously associated with specific phenotypes, such as increased virus binding to α2–3 and α2–6 receptors (Appendix).

**Figure 2:**
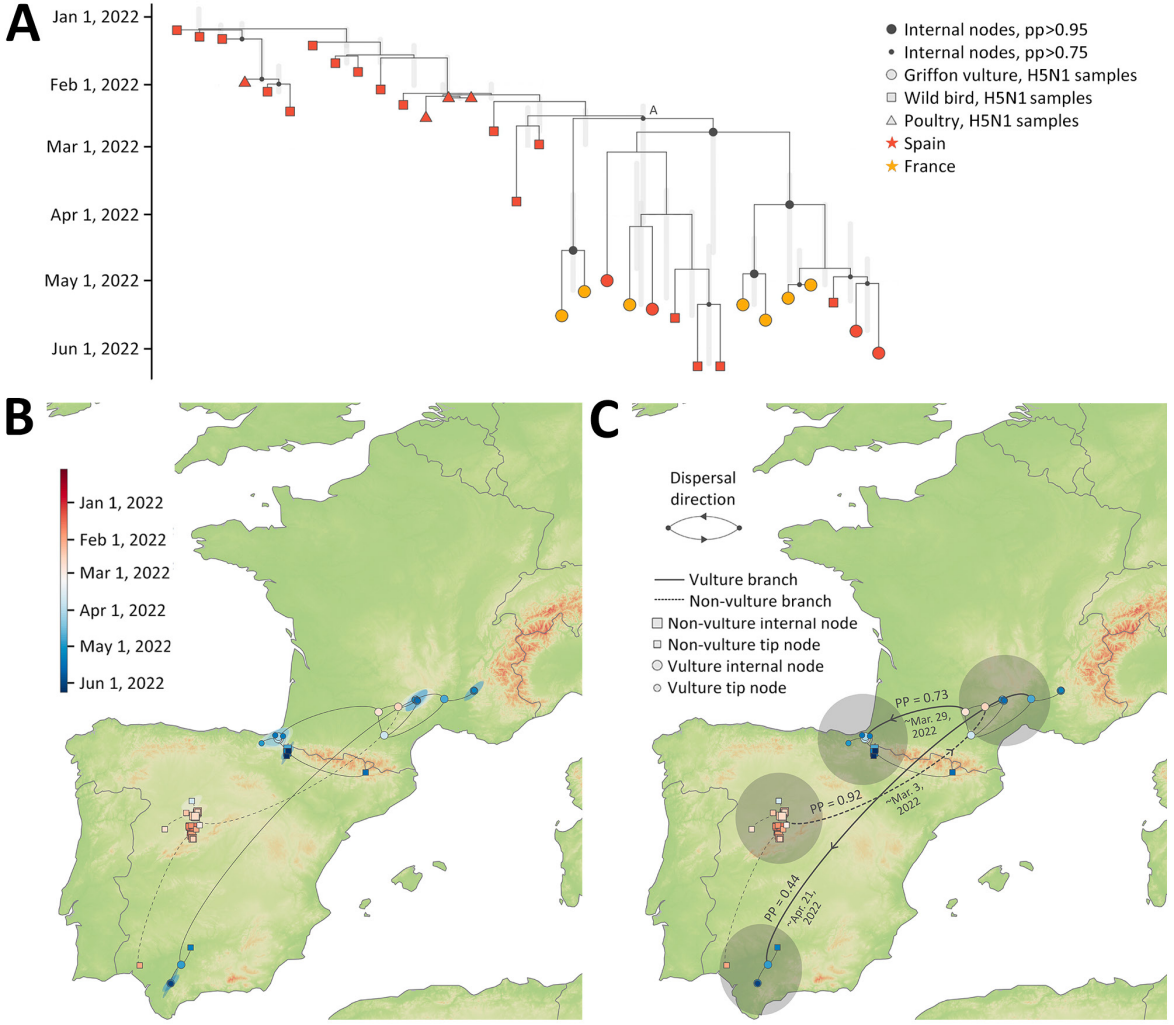
Phylogenetic and phylogeographic analysis conducted in study of multidisciplinary tracking of highly pathogenic avian influenza A(H5N1) outbreak in griffon vultures (*Gyps fulvus*), southern Europe, 2022. A) Maximum clade credibility (MCC) tree obtained from the time-scaled phylogenetic analysis based on genetic sequences of the hemagglutinin gene segment collected from H5N1 virus–infected birds during November 8, 2021–September 1, 2022, in Spain and France. Vertical light gray bars reflect 95% highest posterior density (HPD) intervals associated with the inferred age of internal nodes. B) Continuous phylogeographic reconstruction of the dispersal history of viral lineages. Specifically, we first report the mapped MCC tree and 80% HPD regions reflecting the uncertainty related to the Bayesian continuous phylogeographic inference; both the MCC tree and HPD regions are based on 1,000 trees sampled from the posterior distribution and colored according to their time of occurrence. Phylogenetic branches associated with the vulture subclade (node A) are displayed as solid lines, whereas dashed lines represent other branches in the clade. C) MCC tree as reported in panels A and B, but this time along the PPs and mean estimates associated with lineage dispersal events that occurred between the 4 main regions involved in the continuous phylogeographic reconstruction. PP, posterior probability.

**Figure 3:**
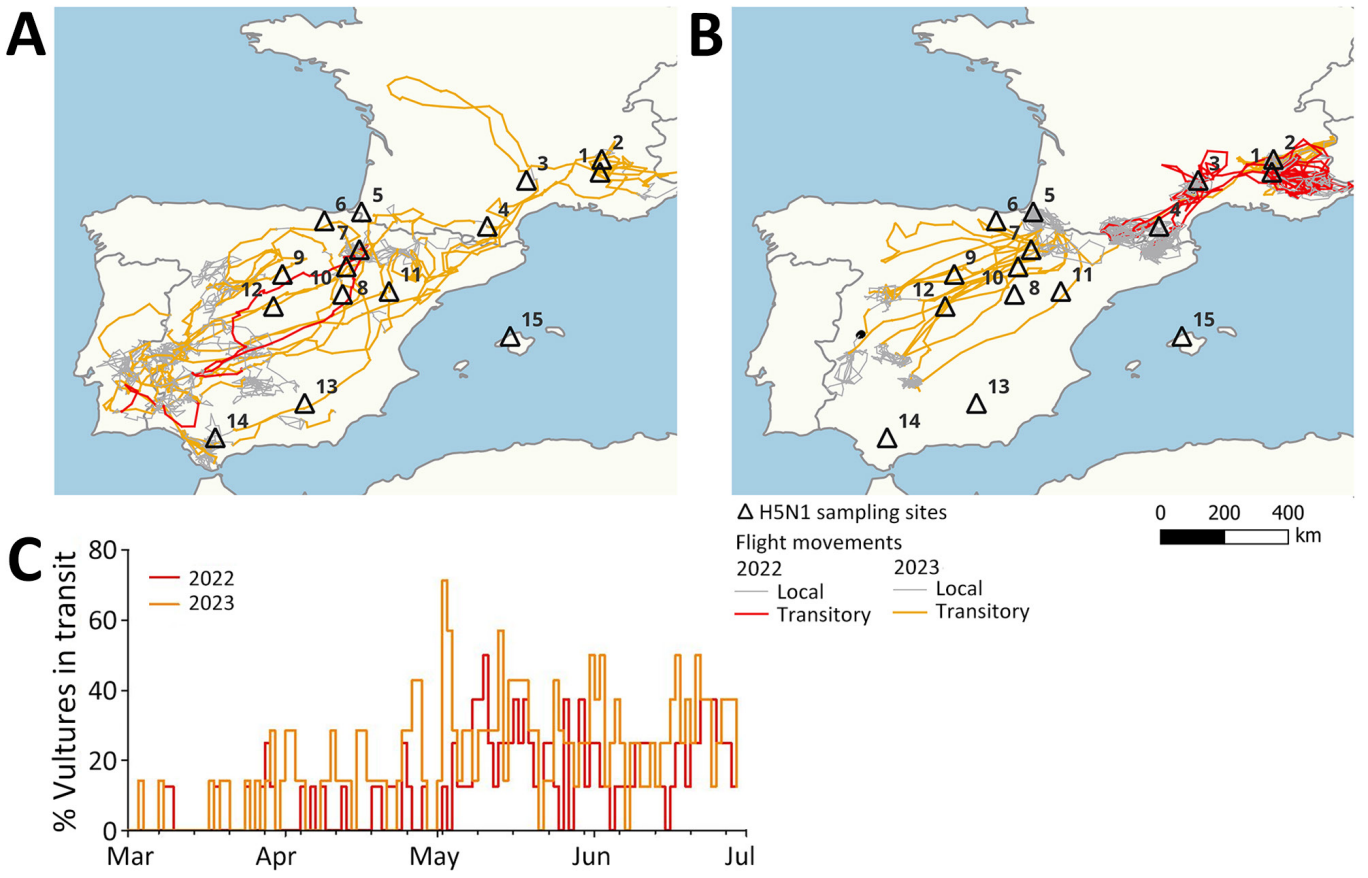
Movements of 16 individual birds during March 1–June 30, 2022, and March 1–June 30, 2023, in study of multidisciplinary tracking of highly pathogenic avian influenza A(H5N1) outbreak in griffon vultures (*Gyps fulvus*), southern Europe, 2022. Immature birds (A) and adult birds (B) show long-range transit movements (rectilinear movements with nocturnal roosts spaced >50 km apart) in red for 2022 and orange for 2023; local movements are shown in gray for both years. C) Timeline of the proportion of individual birds (all ages pooled) in transit every day in 2022 and 2023.

We conducted continuous phylogeographic analysis on this vulture-associated clade and revealed that nearly all (11 of 12) griffon vulture sequences clustered together in a distinct clade (Figure 2, panel A, node A). That clade is supported (posterior probability = 0.84) and contains vulture sequences from both Spain and France. Of note, the only other sequences in this clade originated from bearded vultures (*Gypaetus barbatus*) and a peregrine falcon (*Falco peregrinus*). This pattern suggests that the birds might have shared a common source of infection because of their scavenging feeding behaviors or that some birds could have been initially infected and subsequently transmitted the virus to others. The median time of the most recent common ancestor of the clade (Figure 3, panel A, node A) was estimated to be March 8, 2022 (95% highest posterior density [HPD] February 10, 2022–April 4, 2022). This clade was nested within a larger clade predominantly composed of wild bird sequences from Spain, including greylag geese (*Anser anser*), white storks (*Ciconia ciconia*), and grey herons (*Ardea cinerea*), but that also includes some poultry sequences, suggesting a likely wild bird origin with spillover events to both poultry and vultures (Figure 2, panel A).

Our continuous phylogeographic reconstruction indicated that the vulture viral lineages likely originated from central Spain (Figure 2, panel B) and substantial spatial dispersal was observed across 4 regions (Figure 2, panel C). That dispersal likely originated from wild birds in central Spain, as suggested by the predominance of wild bird sequences in the parent clade, with subsequent spillover events to both poultry and vultures (Figure 2, panel A) and >1 dispersal event toward Massif Central in early March (median date March 3, 2022; 95% HPD February 7, 2022–April 4, 2022; posterior probability = 0.92). Subsequently, lineages spread to the Pyrenees by the end of March (median date March 29, 2022 [95% HPD March 4, 2022–April 20, 2022]; posterior probability = 0.73) and to southern Spain by the end of April (median date April 21, 2022 [95% HPD March 28, 2022–May 4, 2022]; posterior probability = 0.44).

One griffon vulture sequence originating from a rehabilitation center in northern Spain (PP150341; site 6 in Figure 1) fell in a distinct position within the phylogenetic tree (Appendix Figure 2) within a clade of predominantly seabird sequences originating from France. This particular PP150341 sequence is positioned near the BB genotype (H5N1-A/Herring_gull/France/22P015977/2022-like), which has been rapidly expanding across Europe since 2022 (*22*).

### Dispersal of HPAI by Griffon Vulture Movements

To examine changes in the movement patterns of griffon vultures during the HPAI outbreak, we focused on the 4-month period of March–June and compared the movements of flying birds during 2022 compared with 2023. From 114 vultures tagged before the start of the outbreak in Spain and France (94 adult and 20 immature birds, none of which were sampled for viral and serologic monitoring), the percentage of immature birds displaying long-range transit movements was significantly larger than the percentage of adult birds (88% immature vs. 24% adult when both years were pooled; χ^2^ = 37.01, degrees of freedom [d.f.] = 1; p<0.001). The proportion of long-range transit movements did not differ significantly between years (χ^2^ = 0.76, d.f. = 1, p = 0.268 for adults; χ^2^ = 0.09, p = 0.610 for immature birds).

We chose 16 vultures (4 immature and 4 adult birds in both 2022 and 2023) that best visually represent long-range transit movements to further investigate the spatial and temporal dimensions of those movements (Figure 3). The DDT were significantly longer for days of transit compared with days of local movements (181.8 + 77.4 km [max 438.2 km] for days of transit compared with 69.0 + 47.5 km [max 272.1 km] for days of local movement) (Appendix Table 8). Regardless of the type of movement, DDT increased significantly (p<0.001) and progressively over the course of the period (shortest in March and longest in June) (Figure 3, panel C). We observed no qualitative differences in transit movement patterns between 2022 and 2023. In transit movements, adults and immature birds traveled at similar speed, but in general adults took more direct trajectories (Figure 3, panel B). Overall, griffon vultures were able to travel between southern Spain and the Pyrenees or between the Pyrenees and the Alps in 1–2 days.

## Discussion

HPAI H5N1 infections were first detected in griffon vultures in southern Europe in spring 2022 in Spain and France. Infection led to central nervous clinical signs as well as reduced activity, immobility at the roost or nest, and death in some adults. Deaths were also recorded in nestlings, either from direct effects of infection or lack of parental care (*19*).

We investigated the origin of infection and assessed the nature and magnitude of viral spread through an integrated analysis of virologic, serologic, genomic, and ecologic data obtained from field sampling in France and Spain. In addition, we evaluated potential sources of exposure, studied population connectivity pathways, and investigated the potential of long-range contamination by movements of infected birds using long-term GPS tracking data.

Serologic results reflected the circulation of H5 HPAI among flying birds of all sampled colonies of griffon vultures. The high ELISA seroprevalence observed in flying birds seems to indicate widespread transmission but high survival within the meta-population, as opposed to the dramatic mortality rates observed in Sandwich terns, northern gannets, and bald eagles (*20*,*23*,*24*). In contrast, ELISA seroprevalence was considerably lower in nestlings; seropositivity was detected in just 1 colony (Appendix Table 1). That lower seroprevalence could be because of a reduced exposure of nestlings or the result of a high mortality rate. Those seropositive nestlings, detected at the same time as H5N1 AIV–mediated deaths of nestlings in neighboring nests, fledged successfully, demonstrating that infected nestlings, as well as infected adults, were able to survive the infection.

The spread of the virus through the griffon vulture populations in Spain and France was very fast; most H5 HPAI PCR–positive dead birds were collected during April–June 2022, and almost all live and dead birds sampled in both countries after that period tested negative for H5 HPAI virus by qRT-PCR. None of the antibody-positive vultures from our sample set tested positive by PCR, suggesting the absence of active HPAI H5N1 circulation after June 2022, which might relate to the clearance of infection by surviving vultures (*25*,*26*). Sandwich terns sampled in 2022 and 2023 showed a similar pattern; seropositivity was detected in adults in the absence of viral shedding (*24*). However, the lack of data regarding persistence of AIV immunity in griffon vultures, which is known to be highly dependent on the species and age of the host, as well as the subtype of virus, does not preclude circulation at a low prevalence or in the absence of clinical signs (*27*,*28*). Presence of H5-specific AIV antibodies as much as a year after the outbreak, albeit at low titers (Appendix Table 2), might be from antibody persistence or reexposure or from exposure to a different H5 AIV. In fact, AIV seropositivity of vultures in Spain to AIV other than H5 before 2022 provides circumstantial evidence that exposure of vultures to AIV could occur occasionally, which contrasts with the common assumption before the H5N1 outbreak that vultures were either not exposed or not susceptible to AIV infection.

Of note, HPAIV-related deaths of bearded vultures (*G. barbatus*) were reported concurrently to the griffon vulture outbreak (SM17). In contrast, no evidence of infection was found in 2 other cooccurring species of vultures, namely cinereous vultures (*Aegypius monachus*) and Egyptian vultures (*Neophron percnopterus*), which also regularly feed alongside griffon vultures (data not shown).

Phylogenetic and phylogeographic analyses of H5 genetic sequences obtained during the HPAI H5N1 outbreak offered key insights into the potential origins and transmission dynamics of viral lineages among griffon vultures. Of note, the inclusion of almost all griffon vultures within the same genetic cluster, despite having been sampled from geographically distant locations in Spain and France, suggests that they could have shared a common source of infection because of their scavenging feeding behavior, or that some could have been initially infected and subsequently transmitted the virus to others.

Those analyses enable us to draw hypotheses regarding the origin of the virus that infected vultures. This virus was genetically close to strains found in greylag geese and other wetland species, such as grey herons and white storks. Griffon vultures rarely forage in wetlands in France, but they do more often in southern Spain, where they can feed on livestock or wild mammal carcasses in marshes (i.e., potentially close to waterbirds) (Appendix Figure 5). Interactions with waterbirds might also have occurred at small waterbodies in southern Spain, where griffon vultures regularly bathe. Contacts could also have occurred in open urban landfills in northern Spain, where griffon vultures regularly feed alongside other wild bird species that are highly susceptible to the virus, such as gulls and white storks (*29*–*32*). Previous studies have shown potential transmission of low pathogenic avian influenza viruses between species frequenting the same landfills (*33*,*34*). Finally, contacts might involve opportunistic carnivorous mammals, such as red foxes (*Vulpes vulpes*), in which deaths from HPAI H5N1 virus have been reported throughout Europe (*35*). Uncertainties remain regarding the specific mechanisms of the griffon vultures’ contamination.

Our phylogenetic and phylogeographic analyses further suggest that the introduction of HPAI in griffon vultures from poultry farms seems unlikely. This conclusion is also supported by the behavior of griffon vultures, which do not typically visit poultry farm premises. Moreover, culled poultry from affected farms are discarded under strict biosecurity regulations, making the contact of griffon vultures with infected dead poultry unlikely. However, we cannot rule out that griffon vultures might have accessed inadequately discarded undiagnosed dead backyard poultry in some regions of Spain that could also have been consumed by gulls and storks, leading to the further spread and detection of the strain in both wild waterbirds and vultures (*31*,*32*).

The limited genetic diversity of the virus observed in griffon vulture populations, contrasting with the wide geographic distribution of infected birds, suggests that the virus spread in the southwestern Europe metapopulation through intraspecific contamination. The ecology of griffon vultures, especially their feeding behavior and their colonial nature, could explain this finding. Dense short-term aggregations during feeding on carcasses or at vulture feeding stations and dumpsites, where hundreds of individual birds congregate, make griffon vultures particularly vulnerable to airborne pathogen transmission (*36*,*37*). Subsequently, movements of infected birds over long distances could easily have contributed to virus dissemination to the whole population (*38*).

Phylogeographic reconstructions reveal a spatial dissemination pattern across 4 distinct regions, originating from central Spain, spreading to France in the Massif central and the Alps, and subsequently disseminating to the western part of the Pyrenees and southern Spain. This finding is coherent with the analysis of telemetric data, which show an overlap in the distributions of several GPS-tagged birds in Spain and France and long-range movements occurring between populations, particularly in spring, concurrent with the 2022 outbreak (Appendix). Such movements were also observed in other years (*39*), and it thus seems unlikely for them to have been triggered by the outbreak, as observed with northern gannets (*23*,*40*,*41*).

The nestling that was found seronegative and without clinical signs but tested positive for HPAI H5 in the vascular feather provides circumstantial evidence for shedding during the presymptomatic period, because shedding from feather follicles has recently been described as an efficient route of HPAI transmission (*42*). Experimental infection of red-legged partridges (*Alectoris rufa*) with an HPAI H7N1 virus evidenced an incubation period of 3 days with shedding from day 1 (*43*), whereas a similar approach evidenced a 5-day incubation period in falcons (*Falco *spp.) experimentally infected with HPAI H5N1 (*44*). Thus, under the hypothesis of viral incubation lasting >3 days, a griffon vulture infected in the Pyrenees would have enough time to reach southern Spain or the French Alps before showing clinical signs and reduced mobility (*19*). As an example, an immature vulture (Imm_FR_JOR) traveled from southern Portugal to the French Alps in 6 full days (Appendix).

The outbreak described in this study appeared to have had only a mild effect in terms of the conservation of griffon vultures. Compared with long-lived seabird populations in which a large proportion of adults died, mortality in griffon vultures mostly affected nestlings and only few adult birds; adult survival is the most sensitive demographic parameter in such a long-lived species (*45*). In addition, the outbreak struck the world’s largest population of griffon vultures, which could withstand such an ephemeral reduction in breeding success. However, even if griffon vulture populations seem able to overcome this HPAI outbreak, they face multiple threats on a global scale, particularly poisoning and persecution (*46*–*48*). The introduction and circulation of a new infectious pathogen could add additional pressure on the population. Unfortunately, the consequences of a population collapse of necrophagous birds could be catastrophic, especially from a sanitary point of view, in that longer persistence of the carcasses they eliminate would increase risk for pathogen persistence and spread in the environment (*17*,*49*).

Despite a likely limited epidemiologic role of griffon vultures in the circulation of HPAI in Spain and France, with very low permeability between griffon vulture populations and poultry farms, the infection of this new compartment raises multiple questions. In particular, this outbreak demonstrates the ability of this virus (and potentially other highly contagious pathogens) to spread rapidly through a population after a single introduction and shows that even a rare event has the potential for devastating effects.

In conclusion, the recent evolution of HPAI H5N1 has led to this pathogen being considered a severe concern for endangered bird species, especially those with colonial and scavenging behavior. Integrating the epidemiology of the virus with the ecology of the host species is key to a better understanding of outbreak dynamics and possible effects on wildlife conservation. More generally, implementing a multidisciplinary approach will be necessary to overcome these new challenges.

Appendix Additional information about multidisciplinary tracking of highly pathogenic avian influenza A(H5N1) outbreak in griffon vultures, southern Europe, 2022
