# Supplementary material for: Multidisciplinary Tracking of Highly Pathogenic Avian Influenza A(H5N1) Outbreak in Griffon Vultures, Southern Europe, 2022
Source: Emerg Infect Dis. 2025 Aug;31(8):1589–99. doi: 10.3201/eid3108.241456 (PMC12309751; doi:10.3201/eid3108.241456)
Supplement: Appendix — Additional information about multidisciplinary tracking of highly pathogenic avian influenza A(H5N1) outbreak in griffon vultures, southern Europe, 2022 [file 24-1456-Techapp-s1.pdf]

Article DOI: https://doi.org/10.3201/eid3108.241456

EID cannot ensure accessibility for supplementary materials supplied by authors. Readers who have difficulty accessing supplementary content should contact the authors for assistance.

Multidisciplinary Tracking of Highly Pathogenic Avian Influenza A(H5N1) Outbreak in Griffon Vultures, Southern Europe, 2022

Appendix

Additional Methods

Study Sites and Sampling

In Spain, adult Griffon vultures were captured with net traps or walk-in traps at vulture feeding stations, while nestlings were captured at the nests. In France, captures were made using walk-in traps at five sites: Basque Country (western Pyrenees, site 5 in Figure 1), Aude (eastern Pyrenees, site 4), Causses (Massif Central, site 3) and Baronnies-Vercors (Alps, sites 1 and 2) (Appendix Table 1).

All individuals were ringed and a subset of vultures were fitted with GPS satellite transmitters. Blood samples, as well as oropharyngeal and cloacal swabs, were collected from live individuals, while vascular feathers were only collected from a subset of individuals in Spain. On dead individuals, vascular feathers and tissues from the main organs (spleen, pancreas, heart, brain, trachea, intestine, lungs, liver) were collected. In the three badly decomposed carcasses from southern Spain (site 14), only bone marrow and vascular feathers were analyzed because no other tissues were available. Appendix Table 2 details the types of samples available for all individuals in the study.

Ethics considerations

Captures and handling of Griffon vultures in Spain was authorized by the ethical committee of the national research council (CSIC) under permits 12_57-2012 and 21_07_2021) and by the regional governments of Andalusia, Navarra, Castilla y Leon, Aragon, Castilla – La Mancha and the Balearic Islands. In France, capture and ringing-tagging was authorized by CRBPO (French national ringing centre, Museum National d'Histoire Naturelle, Paris) under license PP345, managed by O. Duriez & R. Nadal. Blood and swab sampling was authorized by the prefectures of Lozère, Drôme and Aude in 2022, and from 2023 onwards, the authorization was embedded into the general ringing licence PP345 and French Ministry of research permit #42722-2023031712222922 v4. Capture and handling was carried out in accordance with international, national, and/or institutional guidelines for the care and ethical use of animals, specifically directive 2010/63/EU and Spanish laws 9/2003 and 32/2007, and RD 53/2013.

Appendix Table 1. Details of sampling sites in France and Spain

ID	Site	Region / province	Area	Country	long	lat
1	Villeperdrix	Baronnies	Alps	FR	5.32	44.43
2	Chamaloc	Vercors	Alps	FR	5.37	44.80
3	Cassagne	Causses	Massif Central	FR	3.25	44.20
4	Quillan	Aude	Pyrenees	FR	2.15	42.91
5	Itxassou	French Basque Country	Pyrenees	FR	−1.39	43.32
6	Gipuzkoa/Araba/Bizkaia	Spanish Basque Country	North Spain	SP	−2.44	43.06
7	Ebro Valley	Navarra	North Spain	SP	−1.45	42.25
8	Moncayo	Aragon	Central Spain	SP	−1.82	41.77
9	Hoces del Rio Riaza	Castilla-Leon	Central Spain	SP	−3.62	41.56
10	High Tagus River	Castilla - La Mancha	Central Spain	SP	−1.92	40.99
11	Sierra de Arcos	Aragon	Central Spain	SP	−0.62	41.07
12	Colmenar Viejo	Madrid	Central Spain	SP	−3.80	40.61
13	Cazorla	Andalucia	South Spain	SP	−2.99	37.92
14	Cadiz	Andalucia	South Spain	SP	−5.51	36.95
15	Sierra Tramontana	Mallorca	Balearic Islands	SP	2.79	39.81

*IDs refer to the numbers shown in Figure 1 in the main article. Latitude and longitudes are in Geographic coordinates in decimal degrees (WGS84).

Appendix Table 2. List of samples and associated information used in the study

Country	Area [site ID]	Date	Individual ages	Type of samples	Sample size	Context
Spain	Central Spain [10]	March 2020	Imm, Ad	Swabs (C) and serum	17	Ecologic study
Spain	South Spain [14]	May-June 2021	Imm, Ad	Vascular feathers and serum	10	Ecologic study
Spain	South Spain [13]	June 2021	Nestlings	Vascular feathers and serum	11	Ecologic study
Spain	North Spain [9]	June 2021	Nestlings	Vascular feathers and serum	10	Ecologic study
Spain	North Spain [7]	June -July 2021	Nestlings	Vascular feathers and serum	16	Ecologic study
Spain	Balearic Islands [15]	June-August 2021	Nestlings	Vascular feathers and serum	3	Ecologic study
Spain	Central Spain [8, 11]	November 2021 (1), April (3), June (1) 2022	Imm, Ad	Carcasses (liver)	5 (1+, April, Ad, Ct: 31.8)	Poisoning investigation / Wildlife Influenza surveillance
Spain	North Spain [6]	December 2021 – October 2022	Imm, Ad	Carcasse (tissues, swabs (OP, C))	7 (1+; Ad; Ct: 28.1)	Wildlife influenza surveillance program (dead vultures)

Country	Area [site ID]	Date	Individual ages	Type of samples	Sample size	Context
Spain	North Spain [6]	February – December 2022	Nestlings, Imm, Ad,	Swabs (OP, C) and/or tissues	13 (1+; Ad; Ct: 26.3)	Wildlife influenza surveillance program (live vultures)
Spain	Central Spain [10]	March 2022	Imm, Ad	Swabs (C) and serum	37	Ecologic study
Spain	Central Spain [12]	April 2022	Unknown	Carcasses (liver)	2	Poisoning investigation / Wildlife Influenza surveillance
Spain	South Spain [13]	May 2022	Nestlings	Vascular feathers and serum	10	Ecologic study
Spain	North Spain [6]	May 2022	Ad	Swabs (OP, C) and/or tissues	3 (1+; Ad; Ct: 34.3)	Outbreak follow-up
Spain	South Spain [14]	May-June 2022	Nestlings	Vascular feathers and serum	12 (1+, Nestling, Ct: 29.5)	Ecologic study
Spain	North Spain [7]	June 2022	Nestlings	Vascular feathers and serum	17	Ecologic study
Spain	South Spain [14]	June 2022	Nestlings	Carcasses (bone marrow, vascular feathers)	4 (2+, Nestlings, Ct: 24.8) #	Wildlife Influenza surveillance
Spain	South Spain [14]	August 2022	Imm	Carcass (bone marrow, vascular feather)	1 (1+, Juvenile, Ct: 30.1)	Wildlife Influenza surveillance
Spain	North Spain [7]	July 2022	Nestlings	Carcasses (tissues, bone marrow, vascular feathers)	3	Wildlife Influenza Surveillance
Spain	Central Spain [11]	December 2022	Imm, Ad	Swabs (O/C) and serum	8	Outbreak follow-up
Spain	North Spain [6]	March – September 2023	Imm, Ad	Swabs (OP, C)	11	Wildlife influenza surveillance program (live vultures)
Spain	Central Spain [8]	April 2023	Imm, Ad	Swabs (O/C) and serum	6	Outbreak follow-up
Spain	North Spain [7]	June 2023	Nestlings	Vascular feathers and serum	8	Ecologic study
Spain	South Spain [14]	June 2023	Nestlings	Vascular feathers and serum	2	Ecologic study
Spain	North Spain [6]	August 2023	Imm	Swabs (OP, C) and/or tissues	2	Wildlife influenza surveillance program (dead vultures)
France	Pyrenees, Massif Central and Alps [1,3,4,5]	April – May 2022	Nestlings, Imm, Ad	Carcasses (tissues, swabs (OP, C), vascular feathers)	11 (11+)	Wildlife influenza surveillance program
France	Pyrenees [5]	June 2022	Imm, Ad	Swabs (C) and serum	36	Ecologic study
France	Alps [1]	June 2022	Imm, Ad	Swabs (C) and serum	17	Ecologic study
France	Pyrenees [4]	July 2022	Imm, Ad	Swabs (C) and serum	49	Ecologic study
France	Massif Central [3]	July 2022	Imm, Ad	Swabs (C) and serum	34	Ecologic study
France	Pyrenees [5]	December 2022	Imm, Ad	Swabs (OP, C) and serum	14	Outbreak follow-up
France	Alps [1,2]	December 2022	Imm, Ad	Swabs (OP, C) and serum	50	Outbreak follow-up
France	Massif Central [3]	November 2022	Imm, Ad	Swabs (OP, C) and serum	35	Outbreak follow-up
France	Alps [1,2]	May – June 2023	Imm, Ad	Swabs (OP, C) and serum	70	Outbreak follow-up

Sampling locations are organized by area and site ID (as named in Table S1). The age of sampled individuals refers to nestlings (1st year, before fledging), immature (Imm, from fledging to 4th year) and adult (Ad, older than 4 y). In the column “Type of samples,” “C” stands for cloacal swabs and “OP” for oropharyngeal swabs. Numbers in brackets followed by “+” in the “Sample size” column refer to H5 HPAIV PCR-positive individuals, the age and the cycle threshold (Ct) value is also included. In the column “Context,” “Wildlife influenza surveillance program” refers to passive surveillance with the screening of dead or weak/sick birds while “Outbreak follow-up” refers to active surveillance with the capture and screening of live birds; “Poisoning Investigation/Wildlife Disease surveillance” refers to cases investigated initially for illegal intentional poisoning and later submitted for passive avian influenza surveillance; “Ecological study” refers to the capture of live birds for population or movement monitoring purposes.
Includes the nestling that was alive and tested positive in vascular feathers in May 2022.

Prevalence Study

In Spain, nucleic acids from oropharyngeal and cloacal swabs were extracted using the commercial kits High Pure RNA isolation Kit (Roche diagnostics, Mannheim, Germany), RNeasy Mini Kit (Qiagen, Hilden, Germany) or Biosprint 96 DNA blood kit (Qiagen, Hilden, Germany). Nucleic acids from feather pulp and tissues were extracted using Tri-reagent (Sigma-Aldrich, Madrid, Spain). The extracted RNA was submitted to generic RT-qPCR targeting the influenza A virus matrix gene (*1*). Amplification and detection were performed on an iQ5 real time detection system (BioRad) with the TaqMan fast virus one step kit (Applied Biosystems, New Jersey, USA) or on a QuantStudio 5 Real-Time PCR System (ThermoFisher Scientific Inc., Waltham, MA) with a TaqMan AgPath-ID One-Step RT-PCR kit (ThermoFisher Scientific Inc., Waltham, MA). Positive samples were further tested with an H5 specific RT-qPCR (*2*). Positive samples were tested for isolation in SPF chicken eggs. As isolation attempts were unsuccessful, RNA extracted from field samples were sent to IHAP virology laboratory (IHAP, ENVT, INRAE, Université de Toulouse, Toulouse, France) for Nanopore sequencing.

In France, nucleic acids from all samples were extracted using the commercial kit ID Gene Mag Fast Extraction Kit (Innovative Diagnostics, Grabels, France). The extracted RNA was screened by RT-qPCR targeting H5 and H7 hemagglutinin genes using the commercial PCR kit ID Gene Influenza H5/H7 Triplex (Innovative Diagnostics, Grabels, France). Amplification and detection were performed on a LightCycler® 96 system (Roche Diagnostics, Basel, Switzerland). Positive samples were submitted for Nanopore sequencing (see Nanopore Sequencing section for details).

Serum was separated from the cell pellet by centrifugation in blood samples and stored at −20°C until analysis.

In Spain, sera were inactivated for 30 minutes at 56°C and tested with a commercial ELISA against AIV targeting the nuclear protein (Ingezim Influenza A, Goldstandard, Madrid Spain). ELISA positive samples were submitted to a hemagglutination inhibition (HI) test against H5 and H7 viruses using primary antigens H5N1 (A/Chicken/Scotland/1969, APHA (VLA Weybridge), UK) and H7N1 (A/Afr. Starling/Eng/983/79, APHA (VLA Weybridge), UK). Samples positive against primary antigens were tested against secondary antigens H5N2

(A/Ost/Den/72420/96) and H7N7 (A/tky/Eng/647/77) to confirm positivity against the H antigen.

In France, sera were tested with the commercial ELISA kit ID Screen® Influenza competitive H5 (Innovative Diagnostics, Grabels, France). ELISA positive sera were submitted to HI test against H5 viruses using the primary antigens H5N8 (A/decoyduck/France/161105a/2016) and H5N3 (A/muscovyduck/France/070090b/2007). HI tests were performed according to the NF U 47–036–1 French norm.

HI samples were categorized positive for an antigen if the titer was above 16.

True seroprevalence was then estimated using the "Estimated true prevalence with an imperfect test" tool with Blaker confidence intervals from the epidemiologic calculator Epitools according to the characteristics of commercial kits specified by their manufacturers and relevant literature (ELISA (France) Se = 0.98, Sp = 0.89/(Spain) Se = 0.97, Sp = 0.98, HI Se = 0.85, Sp = 0.99) (*3*–*5*).

Nanopore Sequencing

Multiplex-PCR Amplification Prior to Oxford Nanopore Sequencing

Hemagglutinin and whole genome amplification were performed using PCR primer pools previously described in Croville et al, 2023 (*6*). The PCR amplifications were performed as follows: extracted RNA was reverse transcribed from 10 µL of RNA using a RevertAid First Strand cDNA Synthesis Kit (Thermofisher, Waltham, USA) with 0.5 µM of specific-primer (MBTuni-12 [5′-ACGCGTGATCAGCAAAAGCAGG-3′]) (*7*). Two PCR assays were combined. First, the variable genomic regions encompassing the cleavage site region were amplified by a primer set designed to anneal the hemagglutinin belonging to clade 2.3.4.4 (H5HP). Second, the eight genomic segments of influenza A virus were targeted in a single reaction using the methodology and the following universal primers from Zhou and Wentworth: MBTuni-12 [5′-ACGCGTGATCAGCAAAAGCAGG] and MBTuni-13 [5′-ACGCGTGATCAGTAGAAACAAGG] (*7*). The PCR reactions were performed in a final volume of 20 µL using the Phusion High–Fidelity DNA Polymerase (ThermoFisher) with 8 µL of primer mix H5HP (composed of 1 µM of each primer) or 0.5 µM of universal primers. The temperature cycle parameters were 98°C for 30 s, followed by 35 cycles (98°C for 10 s, 58°C for

20 s, and 72°C for 3 min) with the final extension at 72°C for 10 min. To assess the PCR amplification products, 5 µL of each PCR product was checked on an agarose gel to roughly estimate the amplicons' sizes. Each sample produced amplicons of sufficient size for submission to nanopore sequencing.

Oxford Nanopore Sequencing

PCR products were pooled and purified with a 1:1 ratio of AMPure XP beads. DNA libraries were prepared from 200 fmol of purified PCR products using an SQK-LSK109 Ligation sequencing kit supplied by ONT (Oxford Nanopore Technologies, Oxford, United Kingdom) associated with an EXPNBD104 (ONT) native barcoding kit for the multiplexing of samples. The pooled DNA library was loaded on a MinION Flow Cell R9.4.1 and was run on a MinION Mk1C device (ONT). High accuracy base-calling was performed in real-time with Guppy (v3.5) embedded in the MK1C software (v19.12.12) with the 'Trim Barcode' option on (ONT).

Sequencing Data Analysis

The quantity and quality of data were checked by looking at the sequencing report generated by MinKNOW 2.0 and using NanoPlot from the NanoPack tool set (*8*). The fastq files were aligned using minimap2 (*9*) and the samtools (*10*) package. The HA and whole-genome consensus sequences were generated using the consensus command of the iVar (*11*) pipeline that embeds the samtools mpileup tool (*12*). The consensus sequences were then manually checked using Bioedit (v7.2.6) and IGV (v2.8.2).

Molecular Markers Analysis

Mutation analysis was performed using the original A/H5N1 Goose/Guangdong reference strain from 1996 (*13*) and the A/Vietnam/1203/2004 reference (used to annotate amino acid positions in the CDC inventory (https://archive.cdc.gov/www_cdc_gov/flu/avianflu/h5n1-genetic-changes.htm). Identified mutations were cross-checked with outputs from the FluMut software (https://github.com/izsvenezie-virology/FluMut).

Continuous Phylogeographic Analysis

To place the HPAI H5N1 vulture viruses in the European context and compile a comprehensive dataset, all available H5N1 genetic sequences of the HA segment were retrieved

from the GISAID database (Global Initiative on Sharing All Influenza Data, https://gisaid.org, accessed on February 8, 2024) (*14*), covering the period from November 8, 2021, to September 1, 2022 (n = 1619). Partial sequences, duplicates and sequences with incomplete metadata (e.g., missing collection dates) were removed. The dataset was further subsampled based on identical sequence per country, species and dates. The final set of sequences (n = 575) (https://doi.org/10.57745/8BW5KE) was integrated with the newly generated sequences from vultures from both Spain and France (n = 8, Appendix Table 7). Sequence alignments were generated using MAFFT v7.49 (*15*) and screened using AliView v1.28 (*16*). Maximum likelihood trees were inferred with raxml-ng (*17*) under the HKY + Γ4 model of nucleotide substitution. Based on this analysis, most of the Griffon vulture sequences (n = 11) were grouped into a distinct clade (bootstrap support = 83%, Appendix Figure 2).

To examine the spatial spread of this vulture-associated clade, a continuous phylogeographic analysis was conducted using BEAST v1.10.4 (*18*) with the BEAGLE library v3.0 for improved computational efficiency (*19*). The nucleotide substitution process was modeled using the HKY + Γ4 parametrization (*20*) and the evolutionary process was modeled using an uncorrelated relaxed molecular clock following a lognormal prior distribution (*21*). The tree prior was specified as a skygrid coalescent model (*22*) and the relaxed random walk (RRW) diffusion model (*23*,*24*) was used to perform the continuous phylogeographic reconstruction. Analyses were run for 700 million steps across three independent Markov chains, with states sampled every 100,000 steps. Convergence and mixing properties were assessed using Tracer v1.7.1 (*25*). After discarding 10% of sampled posterior trees as burn-in, the maximum clade credibility (MCC) tree was constructed and annotated using TreeAnnotator v1.10.4 (*18*). The spatiotemporal information embedded within the posterior trees was extracted and visualized using the “seraphim” package in R v4.0.2 (*26*,*27*).

GPS Tracking of Griffon Vultures in Western Europe

Adult (n = 272) and fledging (n = 123) Eurasian Griffon vultures were GPS-tagged in six regions of the Iberian Peninsula and southern France, covering the whole range of Griffon vultures in western Europe (Appendix Table 3, Appendix Figures 3 and 4). Adult birds, identified by plumage features (*28*,*29*), were captured in baited places by means of cannon-nests and walk-in

traps (aviaries) (*30*). Fledglings were captured at between 75 and 110 days old (fledging age c. 120 days).

All birds were tagged with a solar-powered 50 g tag (Ornitela model OT-50 and Ecotone model GRIFFON) or an 85 g e-obs tags. Tags were attached by means of backpack harnesses (Spain) or leg-loop harnesses (France) with the total weight of the system between 1%–2.5% of the total weight of the bird (*31*). All data are stored in the Movebank online database (Appendix Table 4).

Tracking periods were equal to or greater than 1 year (Appendix Table 3). Device configuration was done on the basis of specific objectives of research projects and dependent on season and day length, providing locations every 1–10 min, more frequently during the warmest and sunniest months. For this study, and to describe the movement patterns of individuals, we used all the available locations for each tracked bird.

Appendix Table 3. GPS-tagged Eurasian Griffon vultures in each studied population

Site ID	Region	Age	Period	Nr. Birds
1,2	FR - Alps	adult	2015–2024	49
		immature	2023–2024	7
3	FR – Massif Central	adult	2010–2024	68
		immature	2010–2024	15
4,5	FR - Pyrenees	adult	2013–2024	76
		immature	2021–2024	12
7	SP – North Spain	adult	2015–2024	37
		immature	2012–2024	40
13	SP - South Spain	adult	2014–2023	30
		immature	2021–2024	24
14	SP - South Spain	adult	2018	12
		immature	2021–2024	25

Site IDs refer to Table S1.

Griffon Vulture Movement Data

For the movement analysis, we examined the movement patterns of Griffon vultures during the 4-month period from 1st March to 29th June in 2022 (corresponding to the H5N1 HPAIV outbreak) and 2023 (used as a control, as no outbreak was detected in vultures). We subsampled all data at hourly intervals and calculated Euclidean distances between each consecutive location. Then we summed these hourly distances to estimate the daily distance traveled (DDT). By a visual inspection, we categorized daily tracks as local movements (if they remained in their usual home range and in the evening returned to a roost located within 50 km from their morning starting point), or as transit movements (a more rectilinear daily track with an evening roost located at a distance >50 km away). We also added a third category of "immobile"

behavior, when birds spent more than 3 days with DDT<10 km, as this was found to be a reliable cue to identify individuals potentially clinically affected by HPAI (*32*).

We first examined the movement patterns of a large sample of 114 individuals (37 and 109 individual vultures in 2022 and 2023, respectively) consisting of 94 adults and 20 immatures, tagged in Spain and France. Individuals were categorized as engaging in transit movements (at least 1 day in transit during the period) and as strictly behaving with local movements. From this, we calculated the proportion of individuals engaging in transit movements and compared whether these proportions varied between 2022 and 2023, and among age classes (immatures <5 years-old vs adults) using χ^2 tests.

Next, we examined the spatial and temporal dimensions of the movements of a subsample of 16 individuals that remained in Europe during this period (4 immatures and 4 adults in each year, 2022 and 2023, Table S4). We used a Generalized Linear Mixed Model to identify the factors affecting the DDT, during days when birds were not classified as "immobile." The factors tested were the type of movement (local vs transit), the age of the individual (adult vs immature), the year of tracking (2022 vs 2023), and the month of tracking (March, April, May, June). We used individuals as random factors.

Appendix Table 4. Summary of tracking data from 16 griffon vulture individuals, tagged as nestling or as adult, in Spain or France

		Number of tracking days in type of movement			Total tracking	Movebank		Tagging	
Individual	Year	Local	Transit	Immobile	days	Study	Animal ID	Date	Country - Site ID - Method
Ad_FR_CIJ	2022	102	4	15	121	a	CAU_2018_Ad_Cyrano_CIJ	02/11/2018	FR - 3 - Aviary
Ad_FR_DRU	2022	89	32	0	121	a	BAR_2020_Ad_DRUg	15/01/2020	FR - 1 - Aviary
Ad_FR_ICZ	2022	119	2	0	121	a	AUD_2021_ad_Calmos_ICZd	19/07/2021	FR - 4 - Aviary
Ad_FR_IED	2022	104	10	7	121	a	AUD_2021_ad_Arcade_IEDd	19/07/2021	FR - 4 - Aviary
Ad_FR_ILX	2023	21	14	0	35	a	VEC_2023_ad_ILXd	26/05/2023	FR - 2 - Aviary
Ad_FR_IMX	2023	96	16	9	121	a	BAR_2022_ad_IMXd	07/12/2022	FR - 1 - Aviary
Ad_FR_JPJ	2023	104	12	5	121	a	PBA_2022_Ad_JPJd	10/05/2022	FR - 5 - Aviary
Ad_FR_JPT	2023	104	17	0	121	a	PBA_2022_Ad_JPTd	10/05/2022	FR - 5 - Aviary
Imm_FR_ICG	2022	104	8	9	121	a	AUD_2021_imm_Junior_ICGd	29/06/2021	FR - 4 - Aviary
Imm_SP_0XL	2022	86	27	8	121	b	0XL	29/06/2021	SP - 7 - Nest
Imm_SP_1O1	2022	109	12	0	121	b	1O1	01/07/2021	SP - 7 - Nest
Imm_SP_1OO	2022	94	23	4	121	b	1OO	01/07/2021	SP - 7 - Nest
Imm_FR_JOR	2023	83	38	0	121	a	PBA_2022_chick_JOR	01/06/2022	FR - 5 - Nest
Imm_SP_0XH	2023	94	27	0	121	b	0XH	29/06/2021	SP - 7 - Nest
Imm_SP_0XW	2023	97	24	0	121	b	0XW	30/06/2021	SP - 7 - Nest
Imm_SP_6RL	2023	93	28	0	121	b	6RL	15/06/2022	SP - 7 - Nest

Site IDs refer to Table S1. Movebank studies are Eurasian Griffon Vulture in France ID_PROG 961 (a) or Griffon vulture-imm-Bardenas Reales (b). Note that only 35 d of tracking data are included for individual “Ad_FR_ILX », since it was only captured and tagged on 26 May, 2023, while all other individuals, having been tagged in previous year(s), had the same number of days of tracking data (121).

Additional Results

H5N1 HPAIV-Induced Neurologic Symptoms

See https://doi.org/10.57745/8BW5KE for the video of an H5N1 HPAIV infected vulture exhibiting central nervous system symptoms (Source: Diputación Foral de Gipuzkoa).

H5N1 HPAIV Outbreak Dynamics

Appendix Table 5. Results of ELISA tests and H5 hemagglutination inhibition (H5-HI) test of ELISA positive serum samples collected in Griffon vultures from 2020 to 2023

				ELISA test			H5-HI test	
Country	Area (site ID)	Date	Individual ages	Type	Number (Positive/Total)	Estimated prevalence % [95%CI]	Number (Positive/Total)	Estimated prevalence % [95%CI]
France	Pyrenees (5)	Summer 2022	Imm, Ad	Anti H5	17/36	42 [24–60]	11/16	35 [20–55]

Country	Area (site ID)	Date	Individual ages	ELISA test			H5-HI test	
				Type	Number (Positive/Total)	Estimated prevalence % [95%CI]	Number (Positive/Total)	Estimated prevalence % [95%CI]
		Autumn 2022	Imm, Ad	Anti H5	8/14	53 [25–78]	4/8	33 [13–64]
	Alps (1, 2)	Summer 2022	Imm, Ad	Anti H5	1/17	0 [0–18]	1/1	6 [0–31]
		Autumn 2022	Imm, Ad	Anti H5	25/50	45 [29–60]	11/24	25 [14–41]
		Summer 2023	Imm, Ad	Anti H5	15/70	12 [3–25]	2/15	2 [0–11]
	Pyrenees (4)	Summer 2022	Imm, Ad	Anti H5	10/49	11 [5–26]	9/10	21 [11–36]
		Summer 2023	Imm, Ad	Anti H5	18/42	37 [21–54]	4/18	10 [3–25]
	Massif central (3)	Summer 2022	Imm, Ad	Anti H5	11/34	25 [9–44]	10/11	34 [19–54]
		Autumn 2022	Imm, Ad	Anti H5	12/35	27 [11–46]	5/12	16 [6–34]
		Summer 2023	Imm, Ad	Anti H5	11/45	15 [4–32]	1/7	1 [0–13]
Spain	Central Spain (10)	Spring 2020	Imm, Ad	Anti AIV	8/17	47 [17–67]	0/8	0 [0–21]
		Spring 2022	Imm, Ad	Anti AIV	4/37	11 [4–28]	0/4	0 [0- 10]
	Andalusia (14)	Summer 2021	Nestlings	Anti AIV	0/10	0 [0–32]	NA	NA
		Summer 2022	Nestlings	Anti AIV	3/14	21 [6–48]	3/3	24 [8–55]
		Summer 2023	Nestlings	Anti AIV	0/2	0 [0–67]	NA	NA
	Andalusia (13)	Summer 2021	Nestlings	Anti AIV	0/11	0 [0–25]	NA	NA
		Summer 2022	Nestlings	Anti AIV	0/10	0 [0–27]	NA	NA
	North Spain (7)	Summer 2021	Nestlings	Anti AIV	0/16	0 [0–18]	NA	NA
		Summer 2022	Nestlings	Anti AIV	0/17	0 [0–21]	NA	NA
		Summer 2023	Nestlings	Anti AIV	0/8	0 [0- 32]	NA	NA
	Central Spain (9)	Summer 2021	Nestlings	Anti AIV	0/5	0 [0- 44]	NA	NA
	Central Spain (8)	Summer 2021	Nestlings	Anti AIV	0/5	0 [0- 44]	NA	NA
		Spring 2023	Imm, Ad	Anti AIV	5/6	83 [44–97]	5/5	100 [57–100]
	Balearic Island (15)	Summer 2021	Nestlings	Anti AIV	0/3	0 [0- 57]	NA	NA
	Central Spain (11)	Autumn 2022	Imm, Ad	Anti AIV	8/8	100 [69–100]	2/8	28 [7–69]

For H5-HI individual results, see details in Table S6. Area and site IDs refer to Table S1. The age of sampled individuals refers to nestlings (1st year, before fledging), immature (Imm, from fledging to 4th year) and adult (Ad, older than 4 y).

Appendix Table 6. Results of hemagglutination inhibition tests on French (H5N8 A/decoyduck/France/161105a/2016) and Spanish (H5N1 A/Chicken/Scotland/1969) Griffon vulture samples

Country	Sample ID	Collection period	Area [site ID]	HI titer
France	TY-7164	Summer 2022	Pyrenees [5]	<4
France	VFPB22_02	Summer 2022	Pyrenees [5]	32
France	TY-7162	Summer 2022	Pyrenees [5]	8
France	TY-7171	Summer 2022	Pyrenees [5]	>16
France	VFPB22_09	Summer 2022	Pyrenees [5]	>16
France	TY-7152	Summer 2022	Pyrenees [5]	64
France	TY-7170	Summer 2022	Pyrenees [5]	4
France	VFPB22_20	Summer 2022	Pyrenees [5]	16
France	VFPB22_21	Summer 2022	Pyrenees [5]	128
France	VFPB22_26	Summer 2022	Pyrenees [5]	>16
France	VFPB22_27	Summer 2022	Pyrenees [5]	16
France	VFPB22_30	Summer 2022	Pyrenees [5]	32
France	VFPB22_32	Summer 2022	Pyrenees [5]	32
France	VFPB22_33	Summer 2022	Pyrenees [5]	8
France	VFPB22_34	Summer 2022	Pyrenees [5]	<4
France	VFPB22_36	Summer 2022	Pyrenees [5]	>16
France	VFAU22_03	Summer 2022	Pyrenees [4]	32
France	VFAU22_14	Summer 2022	Pyrenees [4]	32
France	VFAU22_16	Summer 2022	Pyrenees [4]	16
France	O48	Summer 2022	Pyrenees [4]	32
France	VFAU22_28	Summer 2022	Pyrenees [4]	<4
France	VFAU22_31	Summer 2022	Pyrenees [4]	16
France	VFAU22_35	Summer 2022	Pyrenees [4]	16
France	VFAU22_37	Summer 2022	Pyrenees [4]	32
France	VFAU22_38	Summer 2022	Pyrenees [4]	64
France	VFAU22_42	Summer 2022	Pyrenees [4]	32
France	VFCAU22_04	Summer 2022	Massif Central [3]	16
France	TY-5743 FDJ	Summer 2022	Massif Central [3]	64
France	TY-5186 CNN	Summer 2022	Massif Central [3]	16
France	VFCAU22_09	Summer 2022	Massif Central [3]	>16
France	1106584	Summer 2022	Massif Central [3]	16
France	VFCAU22_15	Summer 2022	Massif Central [3]	8
France	TY-4497 JGV	Summer 2022	Massif Central [3]	16
France	VFCAU22_23	Summer 2022	Massif Central [3]	16
France	TY-2867 JCQ	Summer 2022	Massif Central [3]	32
France	VFCAU22_29	Summer 2022	Massif Central [3]	32
France	VFCAU22_30	Summer 2022	Massif Central [3]	16
France	VFBAR22_07	Summer 2022	Alps [1]	32
France	TY7014 CGJ	Autumn 2022	Massif Central [3]	<4
France	TY5140 JHC	Autumn 2022	Massif Central [3]	8
France	TY5767 IDW	Autumn 2022	Massif Central [3]	32
France	1103691	Autumn 2022	Massif Central [3]	32
France	1107854	Autumn 2022	Massif Central [3]	32
France	VF CAU DEC22 05	Autumn 2022	Massif Central [3]	16
France	TY6852 IEL	Autumn 2022	Massif Central [3]	4
France	TY4379 JHQ	Autumn 2022	Massif Central [3]	4
France	TY6121 HFT	Autumn 2022	Massif Central [3]	<4
France	TY2948 JFQ	Autumn 2022	Massif Central [3]	>16
France	VF CAU DEC22 16	Autumn 2022	Massif Central [3]	4
France	VF CAU DEC22 19	Autumn 2022	Massif Central [3]	8
France	BAR DEC22 02	Autumn 2022	Alps [1]	<4
France	BAR DEC22 06	Autumn 2022	Alps [1]	>16
France	BAR DEC22 09	Autumn 2022	Alps [1]	<4
France	BAR DEC22 10	Autumn 2022	Alps [1]	>16
France	BAR DEC22 12	Autumn 2022	Alps [1]	<4
France	BAR DEC22 13	Autumn 2022	Alps [1]	<4
France	TY5445 FTD	Autumn 2022	Alps [1]	4
France	TY4994 DVG	Autumn 2022	Alps [1]	<4
France	TY4279 IMD	Autumn 2022	Alps [1]	<4
France	BAR DEC22 21	Autumn 2022	Alps [1]	4
France	BAR DEC22 22	Autumn 2022	Alps [1]	>16
France	BAR DEC22 25	Autumn 2022	Alps [1]	32
France	BAR DEC22 27	Autumn 2022	Alps [1]	<4
France	BAR DEC22 30	Autumn 2022	Alps [1]	>16
France	BAR DEC22 32	Autumn 2022	Alps [1]	>16
France	BAR DEC22 34	Autumn 2022	Alps [1]	>16
France	BAR DEC22 37	Autumn 2022	Alps [1]	4
France	BAR DEC22 40	Autumn 2022	Alps [1]	32
France	1108455	Autumn 2022	Alps [1]	16

Country	Sample ID	Collection period	Area [site ID]	HI titer
France	BAR DEC22 46	Autumn 2022	Alps [1]	4
France	BAR DEC22 47	Autumn 2022	Alps [1]	4
France	TY6980 IMU	Autumn 2022	Alps [1]	8
France	TY6986 IMV	Autumn 2022	Alps [1]	32
France	TY2317 IML	Autumn 2022	Alps [1]	16
France	TY7154	Autumn 2022	Pyrenees [5]	<4
France	TY6595	Autumn 2022	Pyrenees [5]	>16
France	TY7153	Autumn 2022	Pyrenees [5]	16
France	TY7174	Autumn 2022	Pyrenees [5]	16
France	TY4960	Autumn 2022	Pyrenees [5]	4
France	TY7166	Autumn 2022	Pyrenees [5]	<4
France	TY7165	Autumn 2022	Pyrenees [5]	8
France	TY7175	Autumn 2022	Pyrenees [5]	16
France	TY7101	Summer 2023	Pyrenees [4]	<4
France	TY7001	Summer 2023	Pyrenees [4]	<4
France	TY7008	Summer 2023	Pyrenees [4]	32
France	TY7003	Summer 2023	Pyrenees [4]	16
France	TY7006	Summer 2023	Pyrenees [4]	16
France	TY7005	Summer 2023	Pyrenees [4]	<4
France	TY7002	Summer 2023	Pyrenees [4]	16
France	TY7016	Summer 2023	Pyrenees [4]	<4
France	VFAUD23–05	Summer 2023	Pyrenees [4]	<4
France	TY7009	Summer 2023	Pyrenees [4]	<4
France	VFAUD23–08	Summer 2023	Pyrenees [4]	<4
France	11513	Summer 2023	Pyrenees [4]	<4
France	ZOO154	Summer 2023	Pyrenees [4]	<4
France	NJ7	Summer 2023	Pyrenees [4]	<4
France	VFAUD23–10	Summer 2023	Pyrenees [4]	<4
France	VFAUD23–11	Summer 2023	Pyrenees [4]	<4
France	VFAUD23–14	Summer 2023	Pyrenees [4]	<4
France	VFAUD23–15	Summer 2023	Pyrenees [4]	<4
France	ICONA 1103123	Summer 2023	Alps [1]	<4
France	TY6535	Summer 2023	Alps [1]	16
France	TY7276	Summer 2023	Alps [1]	<4
France	VFVEC23–02	Summer 2023	Alps [1]	<4
France	VFVEC23–06	Summer 2023	Alps [1]	<4
France	VFVEC23–07	Summer 2023	Alps [1]	16
France	VFVEC23–10	Summer 2023	Alps [1]	<4
France	VFVEC23–14	Summer 2023	Alps [1]	8
France	VFVEC23–18	Summer 2023	Alps [1]	<4
France	VFVEC23–20	Summer 2023	Alps [1]	<4
France	VFVEC23–23	Summer 2023	Alps [1]	<4
France	ICONA 1108455	Summer 2023	Alps [1]	4
France	TY5084 FYP	Summer 2023	Alps [1]	<4
France	VFBAR23–19	Summer 2023	Alps [1]	<4
France	VFBAR23–22	Summer 2023	Alps [1]	8
France	VFCAU23–03	Summer 2023	Massif Central [3]	<4
France	VFCAU23–06	Summer 2023	Massif Central [3]	16
France	VFCAU23–08	Summer 2023	Massif Central [3]	<4
France	VFCAU23–13	Summer 2023	Massif Central [3]	<4
France	VFCAU23–34	Summer 2023	Massif Central [3]	<4
France	VFCAU23–35	Summer 2023	Massif Central [3]	<4
France	VFCAU23–44	Summer 2023	Massif Central [3]	<4
Spain	34T	Winter 2020	Central Spain [10]	<4
Spain	MM2	Winter 2020	Central Spain [10]	<4
Spain	34W	Winter 2020	Central Spain [10]	<4
Spain	MM5	Winter 2020	Central Spain [10]	<4
Spain	MM7	Winter 2020	Central Spain [10]	<4
Spain	34V	Winter 2020	Central Spain [10]	<4
Spain	34X	Winter 2020	Central Spain [10]	<4
Spain	352	Winter 2020	Central Spain [10]	<4
Spain	IA22/633	Spring 2022	South Spain [13]	128
Spain	IA22/637	Spring 2022	South Spain [13]	512
Spain	IA22/650	Spring 2022	South Spain [13]	512
Spain	IA22/1715	Autumn 2022	Central Spain [11]	16
Spain	IA22/1717	Autumn 2022	Central Spain [11]	<4
Spain	IA22/1718	Autumn 2022	Central Spain [11]	8
Spain	IA22/1719	Autumn 2022	Central Spain [11]	8
Spain	IA22/1720	Autumn 2022	Central Spain [11]	4
Spain	IA22/1721	Autumn 2022	Central Spain [11]	64
Spain	IA22/1722	Autumn 2022	Central Spain [11]	4

Country	Sample ID	Collection period	Area [site ID]	HI titer
Spain	357	Winter 2022	Central Spain [10]	<4
Spain	35R	Winter 2022	Central Spain [10]	<4
Spain	36C	Winter 2022	Central Spain [10]	<4
Spain	36L	Winter 2022	Central Spain [10]	<4
Spain	IA23/878	Spring 2023	Central Spain [8]	128
Spain	IA23/879	Spring 2023	Central Spain [8]	16
Spain	IA23/880	Spring 2023	Central Spain [8]	8
Spain	IA23/881	Spring 2023	Central Spain [8]	16
Spain	IA23/882	Spring 2023	Central Spain [8]	16
Spain	IA23/883	Spring 2023	Central Spain [8]	16

Area and site IDs refer to Table S1.

Origin and Spread of Infection

Appendix Table 7. Details of the 12 genetic sequences of the HA gene segment from H5N1 HPAIV infected Griffon vultures sampled in Spain and France between November 8, 2021 and September 1, 2022

Accession number	Sequence name	Age	Country	Location [site ID]	Date	Sample type	Context	Sequencing laboratory
PP150340	A/Griffon vulture/Spain/22–04305–001–08/2022	Adult	Spain	Azpeitia [6]	01/05/2022	Brain	Wild	NEIKER
PP150341	A/Griffon vulture/Spain/22–04495–049–00/2022	Adult	Spain	Gipuzkoa [6]	10/05/2022	Oropharyngeal swab	Captive	NEIKER
PP150342	A/Griffon vulture/Spain/22–04643–001–03/2022	Adult	Spain	Irun ([6]	14/05/2022	Brain	Wild	NEIKER
PP150343	A/Griffon vulture/Spain/22–638/2022	Nestling	Spain	Puerto Serrano [14]	24/05/2022	Feather	Wild	IREC
PP150344	A/Griffon vulture/Spain/22–673/2022	Nestling	Spain	Puerto Serrano [14]	03/06/2022	Feather	Wild	IREC
EPI_ISL_14722960	A/vulture/France/22P018210/2022	Nestling	France	Villeperdrix [1]	09/05/2022	Cloacal swab	Wild	ANSES
EPI_ISL_18445706	A/Vulture/France/22P018214/2022	Unknown	France	Villeperdrix [1]	03/05/2022	Unknown	Wild	ANSES
EPI_ISL_18445712	A/Vulture/France/22P016346/2022	Unknown	France	Saint Pierre des Tripiers [3]	17/05/2022	Unknown	Wild	ANSES
EPI_ISL_18445716	A/Vulture/France/22P016720/2022	Unknown	France	Millau [3]	06/05/2022	Unknown	Wild	ANSES
PP191108	A/Griffon vulture/France/LAB22428/2022	Unknown	France	Ainhoa [5])	12/05/2022	Feather	Wild	IHAP
PP191109	A/Griffon vulture/France/LAB22440/2022	Adult	France	Veyreau [3]	19/05/2022	Feather	Wild	IHAP
PP191110	A/Griffon vulture/France/LAB22443/2022	Adult	France	Veyreau [3]	12/05/2022	Trachea	Wild	IHAP

Sequences from NEIKER, IREC and IHAP were generated in this study. Site IDs refer to Table S1.

Spatial Distribution of H5N1 HPAIV Outbreaks and Cases in Poultry, Wild Birds and Vultures

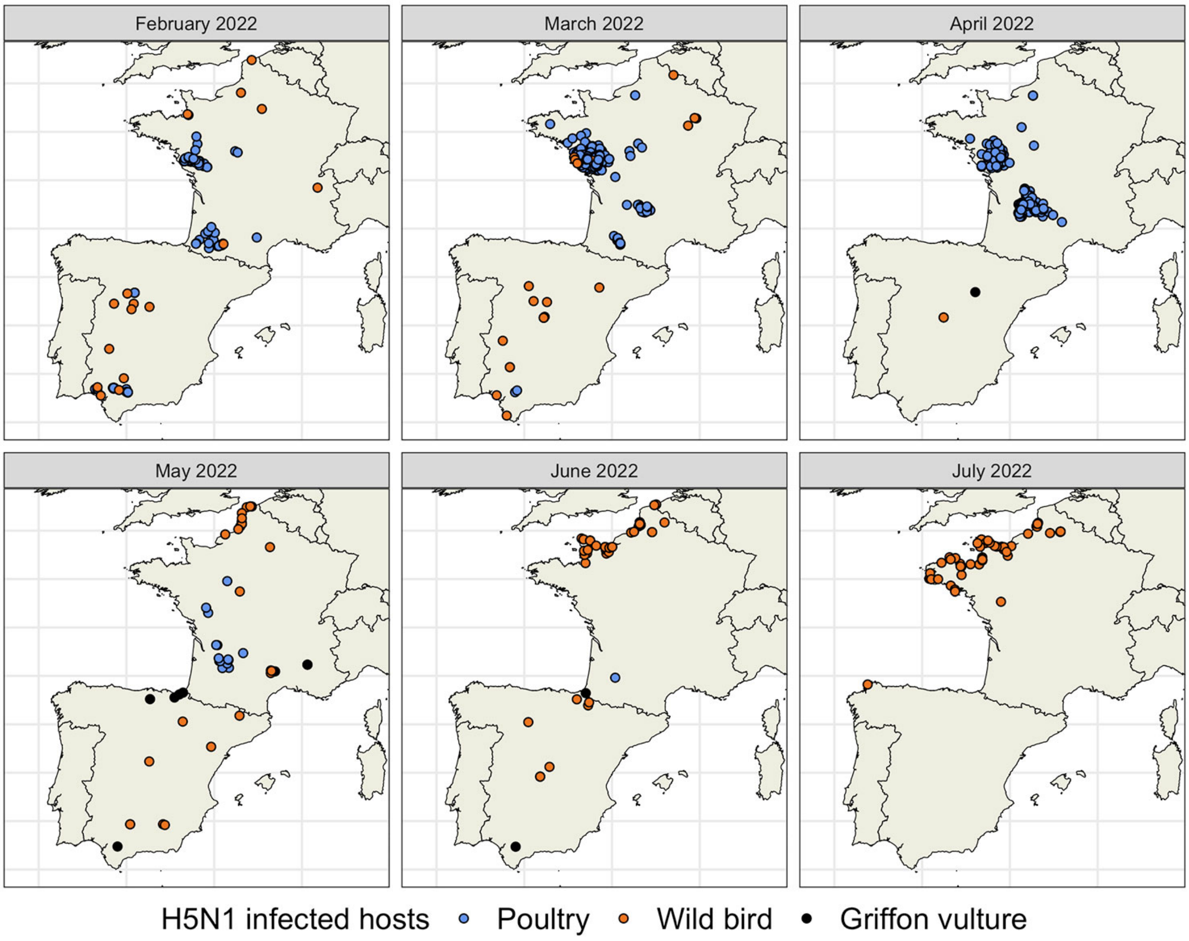

Appendix Figure 1. Spatial distribution of H5N1 HPAIV outbreaks and cases in poultry, wild birds and Griffon vultures reported in France and Spain from February 2022 to July 2022 (source: empres-i). During this period, a total of 14 Griffon vulture cases were reported. Specifically, one was reported in Spain during April, six in France and five in Spain during May, and one in France and one in Spain during June.

Maximum Likelihood Tree Estimated from H5N1 Genetic Sequences of the HA Gene Segment

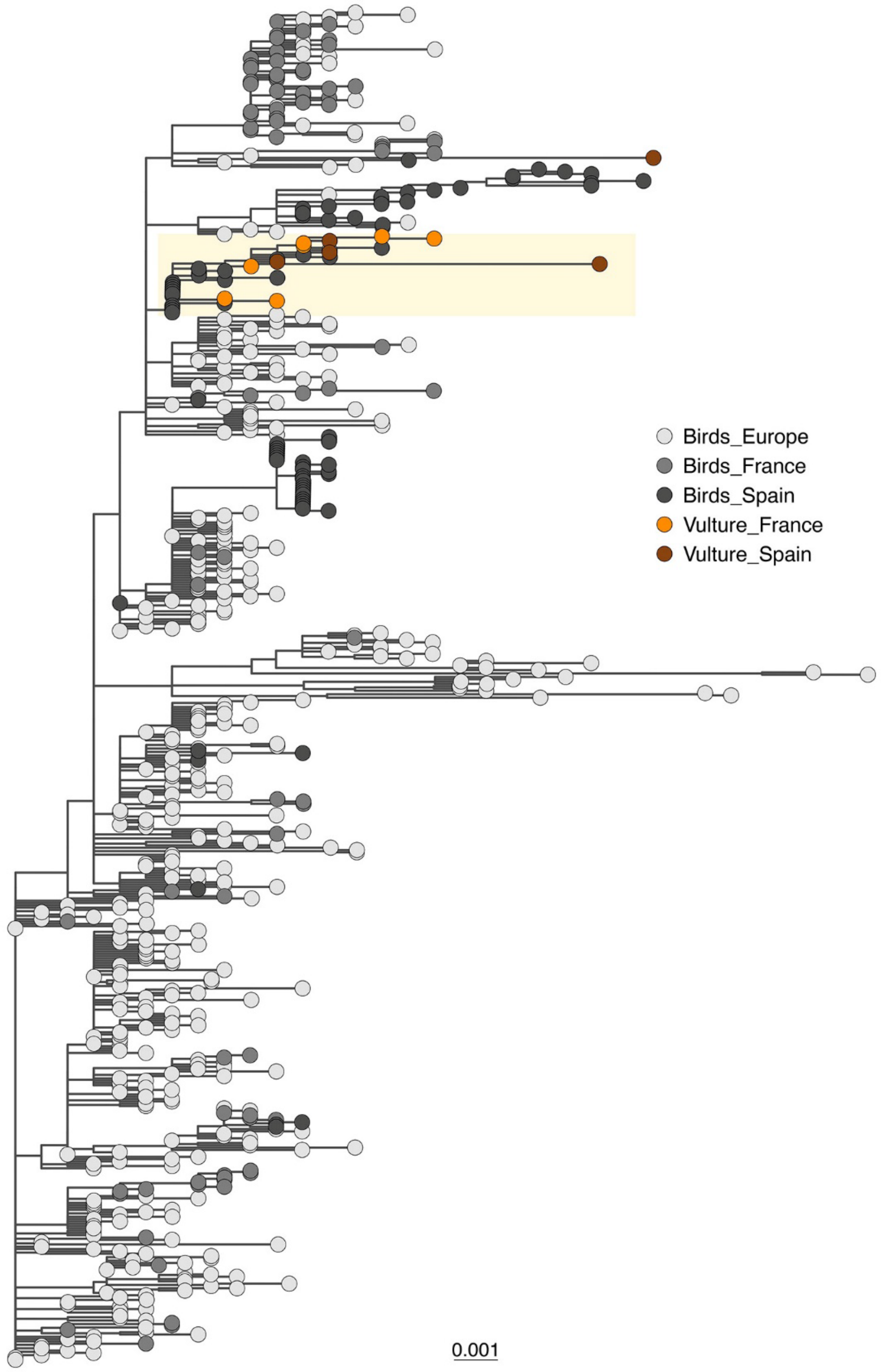

Appendix Figure 2. Maximum likelihood tree estimated from H5N1 genetic sequences of the HA gene segment from vultures in Spain and France and other birds in Europe, Spain and France, from November 8, 2021 to September 1, 2022 (n = 583). Tip labels are colored according to the type of host and origin. The yellow shaded rectangle indicates the clade of interest for the continuous phylogeographic analysis.

Molecular Markers Analysis

See https://doi.org/10.57745/8BW5KE for the molecular markers analysis outputs.

GPS Tracking of Griffon Vultures in Western Europe

The movement patterns revealed long-range movements of vultures between southern Spain, the Pyrenees and the French Alps. Adult birds of all populations (except Alps) regularly visit the same areas in southwestern Iberia (Extremadura and western Andalusia) (Appendix Figure 3). This pattern of movement has already been highlighted in previous studies with smaller numbers of individuals and populations studied, and seems to be conditioned on the high food availability offered by the savannah-like (Dehesa) systems of the southwestern Iberian Peninsula (*33*). As this and other studies have shown (*34*), movements of birds, even in the case of active breeders, can occur in a matter of days between breeding areas separated by distances of more than 500 km, such as northern Iberia and Andalusia. In addition, while some immature birds traveled to Africa, within Europe they also gathered in the same regions of Iberia as adult birds (Appendix Figure 4).

For the population of Cadiz (southern Spain, site 14 in Figure 1 in the main article), to determine the use made by vultures of the Guadalquivir marshes (Doñana), we selected points with a speed of less than 0.35 m/s between 10.00 and 16.00 hours (UTC). We found that vultures nesting in Cadiz regularly stopped in the Doñana marshes during the central hours of the day (Appendix Figure 5). It can be assumed that some of these stops were almost certainly associated with foraging activities (*35*). It has been known for decades that Griffon vultures and other necrophagous birds consume the remains of livestock grazing in the marshlands, as well as the carcasses of wild ungulates and, occasionally, waterfowl (*36*,*37*).

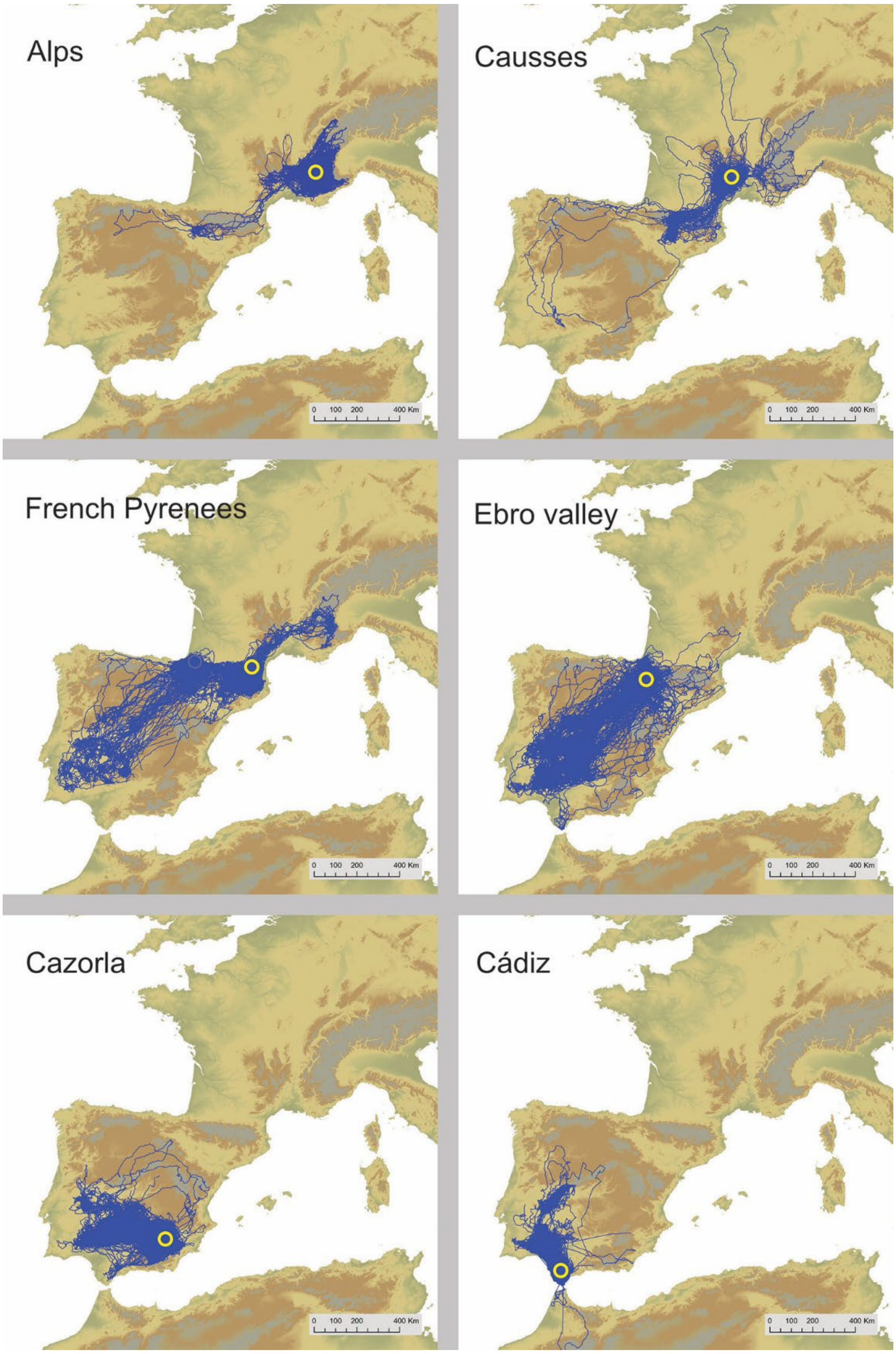

Appendix Figure 3. Tracks of GPS-tagged adult Eurasian Griffon vultures in studied populations of Spain and France from 2010 to 2024. Western and eastern French Pyrenean populations (sites 4 and 5 in Figure 1) were merged. The yellow circles indicate the tagging areas in each case.

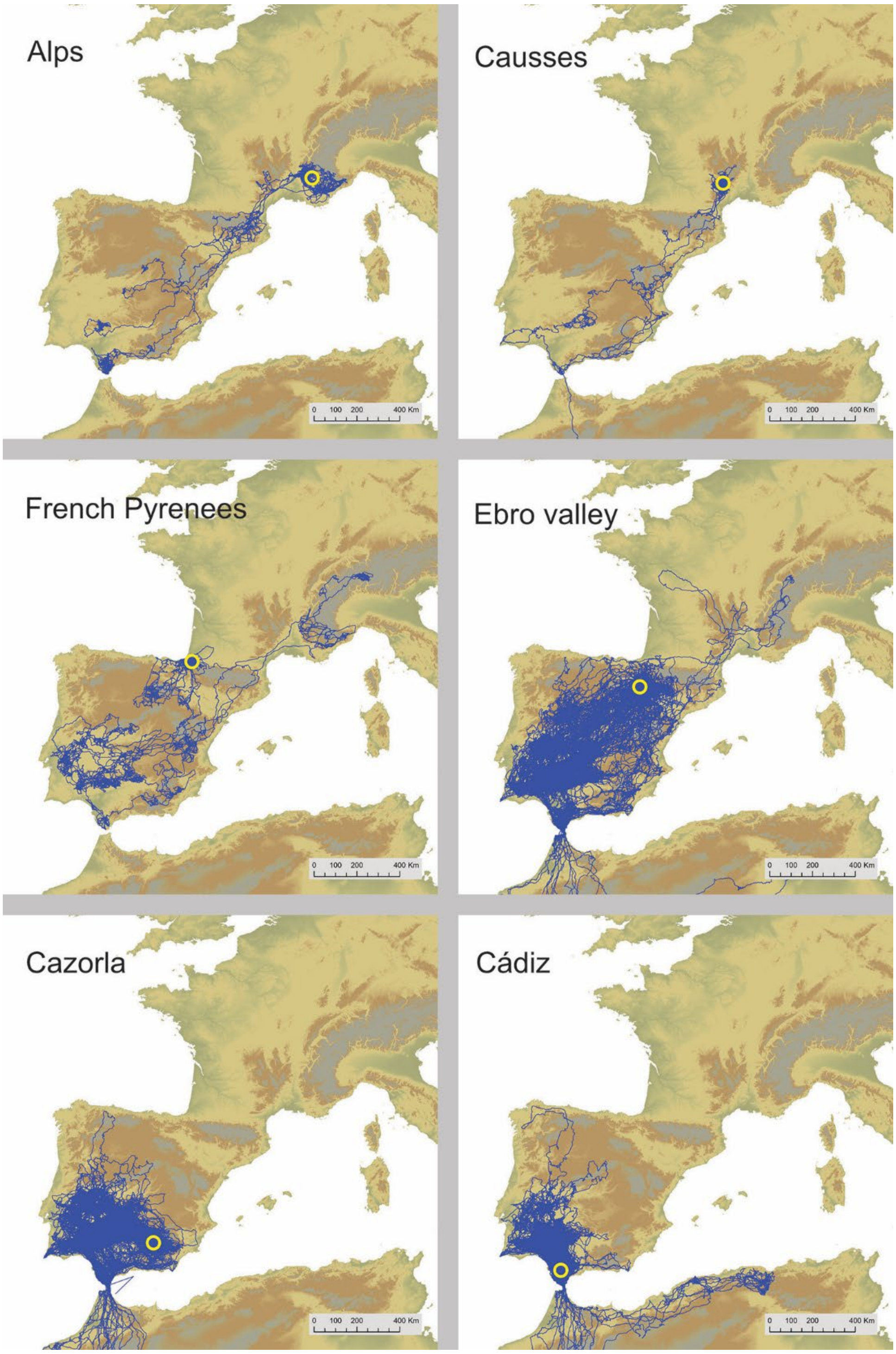

Appendix Figure 4. Tracks of GPS-tagged immature (one calendar year or younger) Eurasian Griffon vultures in studied populations of Spain and France from 2010 to 2024. Western and eastern French Pyrenean populations (sites 4 and 5 in Figure 1) were merged. The yellow circles indicate the tagging areas in each case.

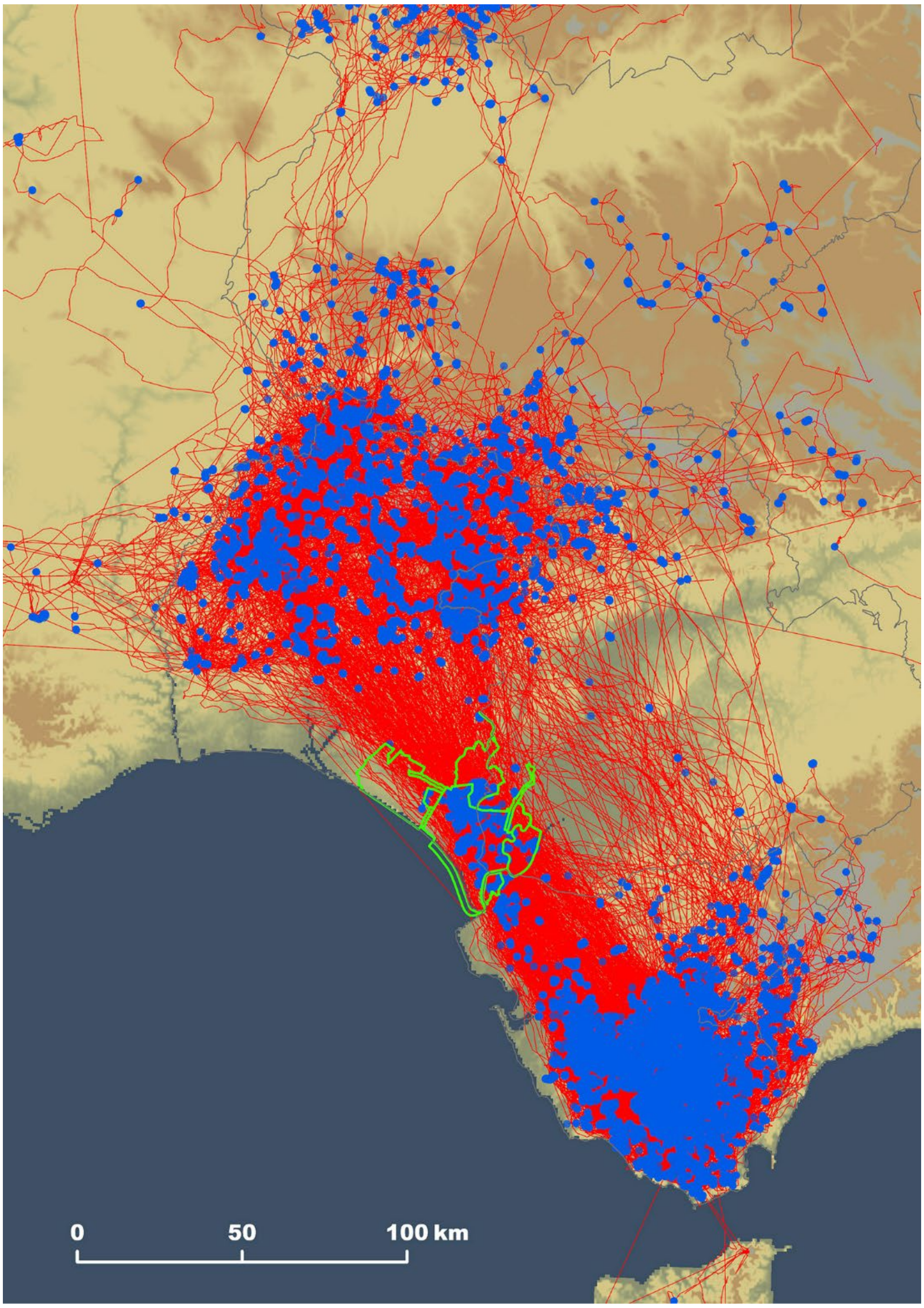

Appendix Figure 5. Movements of GPS-tracked adult Eurasian Griffon vultures breeding in the Cádiz mountains (site 14, Andalusia; yellow circle). Movements (red lines) and stopping points (speed <0.35 m/s) during central hours of the day (10.00–16.00 h UTC) are shown. The boundaries of the Doñana protected area are represented by green lines. The marsh area corresponds to the southernmost protected area.

Griffon Vultures Movement Analysis

Satellite telemetry of 8 tagged individuals revealed long-range movements of vultures across France and Spain in spring 2022 and 2023. Three immatures, in their second calendar

year, tagged as nestlings in northern Spain (Ebro Valley, site 7) traveled from southwestern Spain (Andalusia and Extremadura provinces) toward the western Pyrenees in May 2022. During the same month, three adults tagged in French Basque Country (site 5) in May 2022 made the reverse trip to southwestern Spain. Two adults tagged in summer 2021 in eastern Pyrenees (site 4) traveled to the Massif Central and to the Alps in May 2022. In June 2022 one of the immatures from Spain (site 7) (0XH) continued its trip to the eastern Pyrenees, Massif Central and the Alps. In June 2023, one immature (JOR) traveled from south Portugal to the Alps in only 6 days.

These data show that vulture populations are highly connected throughout France and Spain, and the movements observed in spring 2022 are likely to explain the rapid spread of the virus in both countries, from the likely first infection in southwestern Spain to the French Pyrenees, Massif Central and the Alps.

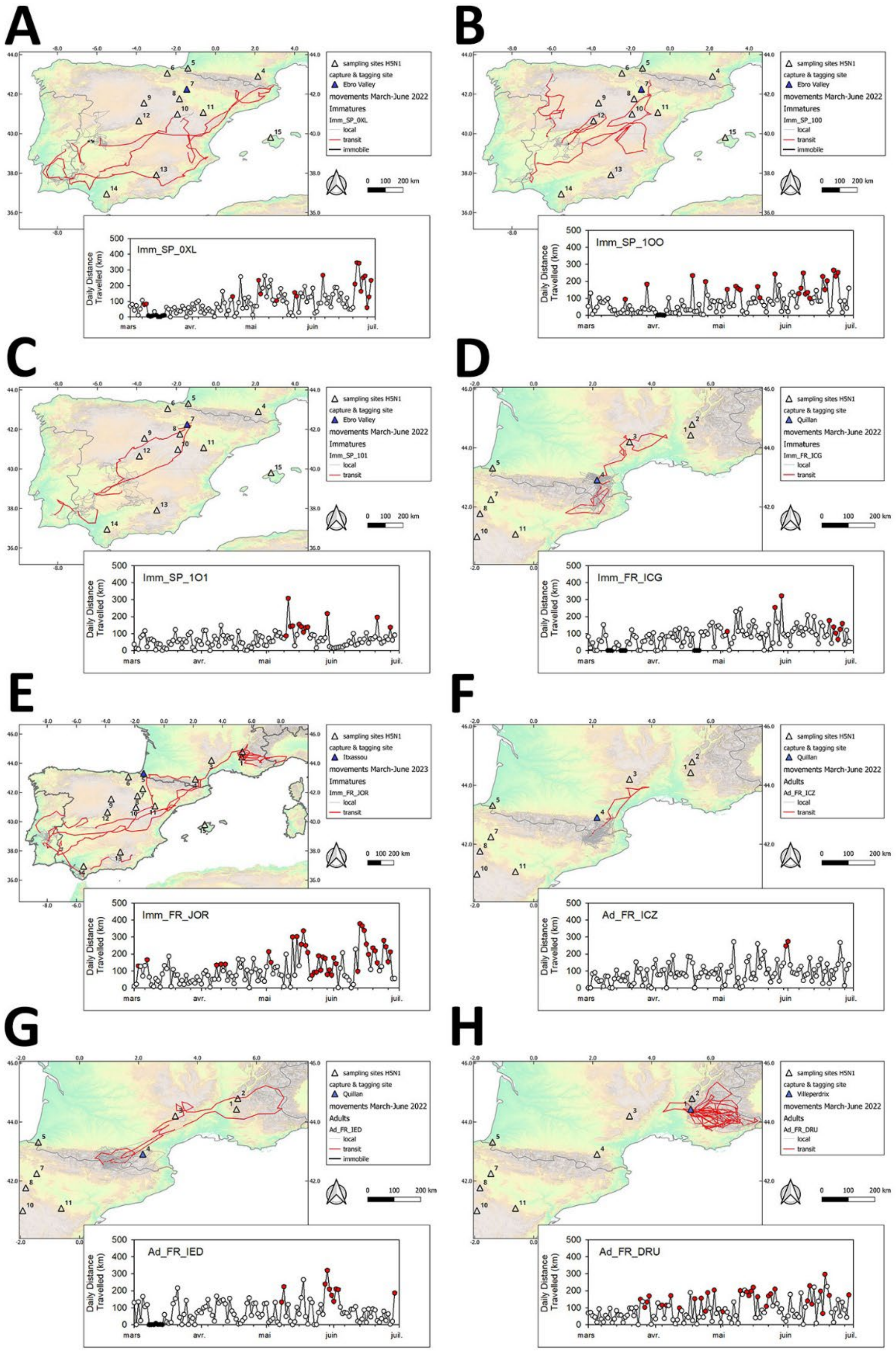

Appendix Figure 6. GPS tracks of 3 adult and 5 immature Griffon vultures tracked in March-June 2022 and 2023. For each individual the map shows the tracks for in transit movements (in red), local movements (in gray) and days of immobility (in black). Sampling sites are indicated by triangles, and the site where the focal individual had been captured and tagged is highlighted in blue. The lower inset shows the daily distance traveled over the 121-day period from 1st March to 29th June 2022/2023, highlighting the days when the individual was in transit (red), in local movement (white) and immobile (black).

Statistics of Griffon Vulture Movements

Appendix Table 8. Summary of GLMM type III tests of fixed effects on dependent variable Daily Distance Traveled (DDT)

Source	Numerator df	Denominator df	F	p-value
Intercept	1	1786.000	5165.983	<0.001
Age	1	1786.000	3.911	.048
Year	1	1786.000	2.185	.140
Movement type	1	1786.000	936.112	<0.001
Month	3	1786.000	25.140	<0.001

DDT was mainly affected by the type of movement (larger during transit than during local movements) and by the month (increasing from March to June), while the effect of age was marginal and the effect of year was not significant.

Appendix Table 9. Summary statistics of daily distance traveled (DDT, in km) according to type of movement and month of the year

Movement type	Month	Nb	Mean	SD	Minimum	Maximum	Percentiles 25	50	75
Local	3	416	53.349	38.459	0.112	215.488	22.638	48.666	77.856
	4	390	70.460	45.386	0.258	256.848	33.090	67.821	98.401
	5	341	75.126	52.369	0.071	272.113	34.649	73.852	103.253
	6	352	79.807	49.982	0.776	267.388	42.813	77.455	105.868
Transit	3	19	124.140	47.507	0.427	219.707	93.690	129.167	152.384
	4	45	175.236	86.220	56.922	432.858	109.728	155.702	225.153
	5	118	181.574	76.508	74.131	438.236	131.293	166.060	215.476
	6	112	194.511	74.608	2.820	416.027	142.536	188.747	241.128

Additional Results in Bearded Vultures

Four carcasses of Bearded vultures were collected between May and June 2022 in Spain (no carcasses were found in France). All data from Bearded vultures were obtained from GISAID and the Laboratorio Central de Veterinaria in Spain.

Carcasses of three nestlings were collected at nests in the Pyrenees and southern Spain. These three sequences (Table S10) were genetically closely related to those found in Griffon vultures (Appendix Figure 2), suggesting that Bearded vultures may have been infected while feeding alongside Griffon vultures, as they commonly do at vulture restaurants. The relatively low number of diagnosed cases in Bearded vultures could be due to their distinct ecology, characterized by solitary and territorial behavior, their much smaller population size and the difficulty of detection of deceased individuals, especially adults, at high altitudes (*38*). Akin to the breeding failure reported for Bald eagles, nesting failure of Bearded vultures in the western Pyrenees increased considerably in 2022, but was not further investigated (data not shown).

The fourth carcass concerned a captive adult individual from Cordoba Zoo. In contrast, its sequence showed close genetic similarity to sequences from other captive wild species, as well as Spanish poultry, suggesting that the local spread of the virus likely originated from poultry farms as raptors may occasionally be fed with chicken carcasses at such premises.

Appendix Table 10. Details of the 4 genetic sequences of the HA gene segment from H5N1 HPAI infected Bearded vultures in Spain collected between November 8, 2021 to September 1, 2022. Site IDs refer to Table S1

Accession number	Sequence name	Age	Country	Location [site ID]	Date	Sample type	Context	Sequencing laboratory
EPI_ISL_18075816	A/bearded_vulture/Spain/2116–1-2022_23VIR6502–1/2022	Nestling	Spain	Oroz-Betelu [7]	07/06/2022	Unknown	Wild	Laboratorio Central de Veterinaria
EPI_ISL_15234656	A/bearded_vulture/Spain/2116–3_22VIR8632–8/2022	Nestling	Spain	Longuida [7]	09/06/2022	Unknown	Wild	Laboratorio Central de Veterinaria
EPI_ISL_13990717	A/Gypaetus_barbatus/Spain/1878–9_22VIR6312–18/2022	Nestling	Spain	Peal de Becerro [13]	11/05/2022	Unknown	Wild	Laboratorio Central de Veterinaria
EPI_ISL_13990718	A/Gypaetus_barbatus/Spain/1956–25_22VIR6312–19/2022	Unknown	Spain	Cordoba (Cordoba)	27/05/2022	Unknown	Captive	Laboratorio Central de Veterinaria

References

1. Munster VJ, Baas C, Lexmond P, Waldenström J, Wallensten A, Fransson T, et al. Spatial, temporal, and species variation in prevalence of influenza A viruses in wild migratory birds. PLoS Pathog. 2007;3:e61. PubMed https://doi.org/10.1371/journal.ppat.0030061

2. Spackman E, Senne DA, Myers TJ, Bulaga LL, Garber LP, Perdue ML, et al. Development of a real-time reverse transcriptase PCR assay for type A influenza virus and the avian H5 and H7 hemagglutinin subtypes. J Clin Microbiol. 2002;40:3256–60. PubMed https://doi.org/10.1128/JCM.40.9.3256-3260.2002

3. Sergent E. EpiTools—epidemiological calculators [cited 2023 Jan 31]. https://epitools.ausvet.com.au/

4. Reiczigel J, Földi J, Ozsvári L. Exact confidence limits for prevalence of a disease with an imperfect diagnostic test. Epidemiol Infect. 2010;138:1674–8. PubMed https://doi.org/10.1017/S0950268810000385

5. Rogan WJ, Gladen B. Estimating prevalence from the results of a screening test. Am J Epidemiol. 1978;107:71–6. PubMed https://doi.org/10.1093/oxfordjournals.aje.a112510

6. Croville G, Walch M, Lèbre L, Silva S, Filaire F, Guérin JL. An amplicon-based nanopore sequencing workflow for rapid tracking of avian influenza outbreaks, France, 2020–2022. Front Cell Infect Microbiol. 2024;14:1257586. **PMID 38318163**

7. Zhou B, Donnelly ME, Scholes DT, St George K, Hatta M, Kawaoka Y, et al. Single-reaction genomic amplification accelerates sequencing and vaccine production for classical and Swine origin human influenza a viruses. J Virol. 2009;83:10309–13. PubMed https://doi.org/10.1128/JVI.01109-09

8. De Coster W, D'Hert S, Schultz DT, Cruts M, Van Broeckhoven C. NanoPack: visualizing and processing long-read sequencing data. Bioinformatics. 2018;34:2666–9. PubMed https://doi.org/10.1093/bioinformatics/bty149

9. Li H. Minimap2: pairwise alignment for nucleotide sequences. Bioinformatics. 2018;34:3094–100. PubMed https://doi.org/10.1093/bioinformatics/bty191

10. Danecek P, Bonfield JK, Liddle J, Marshall J, Ohan V, Pollard MO, et al. Twelve years of SAMtools and BCFtools. Gigascience. 2021;10:giab008. PubMed https://doi.org/10.1093/gigascience/giab008

11. Grubaugh ND, Gangavarapu K, Quick J, Matteson NL, De Jesus JG, Main BJ, et al. An amplicon-based sequencing framework for accurately measuring intrahost virus diversity using

PrimalSeq and iVar. Genome Biol. 2019;20:8. PubMed https://doi.org/10.1186/s13059-018-1618-7

12. Li H. A statistical framework for SNP calling, mutation discovery, association mapping and population genetical parameter estimation from sequencing data. Bioinformatics. 2011;27:2987–93. PubMed https://doi.org/10.1093/bioinformatics/btr509

13. Harfoot R, Webby RJ. H5 influenza, a global update. J Microbiol. 2017;55:196–203. PubMed https://doi.org/10.1007/s12275-017-7062-7

14. Shu Y, McCauley J. GISAID: global initiative on sharing all influenza data - from vision to reality. Euro Surveill. 2017;22:30494. PubMed https://doi.org/10.2807/1560-7917.ES.2017.22.13.30494

15. Katoh K, Standley DM. MAFFT multiple sequence alignment software version 7: improvements in performance and usability. Mol Biol Evol. 2013;30:772–80. PubMed https://doi.org/10.1093/molbev/mst010

16. Larsson A. AliView: a fast and lightweight alignment viewer and editor for large datasets. Bioinformatics. 2014;30:3276–8. PubMed https://doi.org/10.1093/bioinformatics/btu531

17. Kozlov AM, Darriba D, Flouri T, Morel B, Stamatakis A. RAxML-NG: a fast, scalable and user-friendly tool for maximum likelihood phylogenetic inference. Bioinformatics. 2019;35:4453–5. PubMed https://doi.org/10.1093/bioinformatics/btz305

18. Drummond AJ, Rambaut A. BEAST: Bayesian evolutionary analysis by sampling trees. BMC Evol Biol. 2007;7:214. PubMed https://doi.org/10.1186/1471-2148-7-214

19. Ayres DL, Cummings MP, Baele G, Darling AE, Lewis PO, Swofford DL, et al. BEAGLE 3: improved performance, scaling, and usability for a high-performance computing library for statistical phylogenetics. Syst Biol. 2019;68:1052–61. PubMed https://doi.org/10.1093/sysbio/syz020

20. Shapiro B, Rambaut A, Drummond AJ. Choosing appropriate substitution models for the phylogenetic analysis of protein-coding sequences. Mol Biol Evol. 2006;23:7–9. PubMed https://doi.org/10.1093/molbev/msj021

21. Drummond AJ, Ho SYW, Phillips MJ, Rambaut A. Relaxed phylogenetics and dating with confidence. PLoS Biol. 2006;4:e88. PubMed https://doi.org/10.1371/journal.pbio.0040088

22. Hill V, Baele G. Bayesian estimation of past population dynamics in BEAST 1.10 using the skygrid coalescent model. Mol Biol Evol. 2019;36:2620–8. PubMed https://doi.org/10.1093/molbev/msz172

23. Lemey P, Rambaut A, Welch JJ, Suchard MA. Phylogeography takes a relaxed random walk in continuous space and time. Mol Biol Evol. 2010;27:1877–85. PubMed https://doi.org/10.1093/molbev/msq067

24. Pybus OG, Suchard MA, Lemey P, Bernardin FJ, Rambaut A, Crawford FW, et al. Unifying the spatial epidemiology and molecular evolution of emerging epidemics. Proc Natl Acad Sci U S A. 2012;109:15066–71. PubMed https://doi.org/10.1073/pnas.1206598109

25. Rambaut A, Drummond AJ, Xie D, Baele G, Suchard MA. Posterior summarization in Bayesian phylogenetics Using Tracer 1.7. Syst Biol. 2018;67:901–4. PubMed https://doi.org/10.1093/sysbio/syy032

26. Dellicour S, Rose R, Faria NR, Lemey P, Pybus OG. SERAPHIM: studying environmental rasters and phylogenetically informed movements. Bioinformatics. 2016;32:3204–6. PubMed https://doi.org/10.1093/bioinformatics/btw384

27. Dellicour S, Rose R, Faria NR, Vieira LFP, Bourhy H, Gilbert M, et al. Using viral gene sequences to compare and explain the heterogeneous spatial dynamics of virus epidemics. Mol Biol Evol. 2017;34:2563–71. PubMed https://doi.org/10.1093/molbev/msx176

28. Duriez O, Eliotout B, Sarrazin F. Age identification of Eurasian Griffon Vultures *Gyps fulvus* in the field. Ring Migr. 2011;26:24–30. https://doi.org/10.1080/03078698.2011.585912

29. Zuberogoitia I, De la Puente J, Elorriaga J, Alonso R, Palomares L, Martínez J. The flight feather molt of Griffon vultures (*Gyps fulvus*) and associated biological consequences. J Raptor Res. 2013;47:292–303. https://doi.org/10.3356/JRR-12-09.1

30. Duriez O, Harel R, Hatzofe O. Studying movement of avian scavengers to understand carrion ecology. In: Olea PP, Mateo-Tomás P, Sánchez-Zapata JA, editors. Carrion ecology and management. Cham: Springer International Publishing; 2019. p. 255–74.

31. Anderson D, Terrace M, Arkumarev V, Bildstein K, Botha A, Bowden C, et al. A practical guide to methods for attaching research devices to vultures and condors. Vulture News. 2020.

32. Duriez O, Sassi Y, Le Gall-Ladevèze C, Giraud L, Straughan R, Dauverné L, et al. Highly pathogenic avian influenza affects vultures' movements and breeding output. Curr Biol. 2023;33:3766–3774.e3. PubMed https://doi.org/10.1016/j.cub.2023.07.061

33. Delgado-González A, Cortés-Avizanda A, Serrano D, Arrondo E, Duriez O, Margalida A, et al. Apex scavengers from different European populations converge at threatened savannah landscapes. Sci Rep. 2022;12:2500. PubMed https://doi.org/10.1038/s41598-022-06436-9

34. Martínez F, Oltra J, Frías Ó, González Del Barrio JL, Pérez-García JM, Carrete M, et al. A long-lasting, distant journey of a male griffon vulture informs on the success of differential parental investment. Ecology. 2024;105:e4226. PubMed https://doi.org/10.1002/ecy.4226

35. Arrondo E, Sebastián-González E, Moleón M, Morales-Reyes Z, María Gil-Sánchez J, Cortés-Avizanda A, et al. Vulture culture: dietary specialization of an obligate scavenger. Proc Biol Sci. 2023;290:20221951. **PMID 37132232**

36. Valverde JA. Structure of a Mediterranean community of terrestrial vertebrates [in Spanish]. 1967 [cited 2024 May 7]. https://digital.csic.es/handle/10261/114370

37. Hiraldo F, Blanco JC, Bustamante J. Unspecialized exploitation of small carcasses by birds. Bird Study. 1991;38:200–7. https://doi.org/10.1080/00063659109477089

38. Margalida A, Jiménez J, Martínez JM, Sesé JA, García-Ferré D, Llamas A, et al. An assessment of population size and demographic drivers of the Bearded Vulture using integrated population models. Ecol Monogr. 2020;90:e01414. https://doi.org/10.1002/ecm.1414
